# Estimation of 100 m root zone soil moisture by downscaling 1 km soil water index with machine learning and multiple geodata

**DOI:** 10.1007/s10661-024-12969-5

**Published:** 2024-08-19

**Authors:** Talha Mahmood, Johannes Löw, Julia Pöhlitz, Jan Lukas Wenzel, Christopher Conrad

**Affiliations:** Department of Geoecology, Institute of Geosciences and Geography, Martin Luther University Halle-Wittenberg, 06120 Halle (Saale), Germany

**Keywords:** Soil water index, Synthetic aperture radar, Random forest downscaling, DEMMIN, Irrigation management

## Abstract

Root zone soil moisture (RZSM) is crucial for agricultural water management and land surface processes. The 1 km soil water index (SWI) dataset from Copernicus Global Land services, with eight fixed characteristic time lengths (*T*), requires root zone depth optimization (*T*_opt_) and is limited in use due to its low spatial resolution. To estimate RZSM at 100-m resolution, we integrate the depth specificity of SWI and employed random forest (RF) downscaling. Topographic synthetic aperture radar (SAR) and optical datasets were utilized to develop three RF models (RF1: SAR, RF2: optical, RF3: SAR + optical). At the DEMMIN experimental site in northeastern Germany, *T*_opt_ (in days) varies from 20 to 60 for depths of 10 to 30 cm, increasing to 100 for 40–60 cm. RF3 outperformed other models with 1 km test data. Following residual correction, all high-resolution predictions exhibited strong spatial accuracy (*R* ≥ 0.94). Both products (1 km and 100 m) agreed well with observed RZSM during summer but overestimated in winter. Mean *R* between observed RZSM and 1 km (100 m; RF1, RF2, and RF3) SWI ranges from 0.74 (0.67, 0.76, and 0.68) to 0.90 (0.88, 0.81, and 0.82), with the lowest and highest *R* achieved at 10 cm and 30 cm depths, respectively. The average RMSE using 1 km (100 m; RF1, RF2, and RF3) SWI increased from 2.20 Vol.% (2.28, 2.28, and 2.35) at 30 cm to 3.40 Vol.% (3.50, 3.70, and 3.60) at 60 cm. These negligible accuracy differences underpin the potential of the proposed method to estimate RZSM for precise local applications, e.g., irrigation management.

**Supplementary Information:**

The online version contains supplementary material available at 10.1007/s10661-024-12969-5.

## Introduction

Soil moisture is a relevant parameter of the surface energy balance and is crucial for environmental applications such as drought monitoring, water resources management, and flood prediction (Babaeian et al., 2019). Soil moisture steers crop production in agricultural water management (Pawar & Khanna, 2018). The depletion of soil moisture can cause conditions in the soil, which hampers crop growth, reduces yield, and poses a threat to food security (Xing et al., 2022). Traditionally, soil moisture is monitored using in situ measurements, which offers accurate estimates of soil moisture with high temporal resolution. However, despite the accuracy, this method is costly and laborious and suffers from low spatial representation (Rasheed et al., 2022). Numerous remote sensing satellite platforms launched in the last decades allowed for supplying the demand for economically feasible soil moisture information at a global scale with a temporal frequency of up to a few days (Prajapati et al., 2018; Zawadzki & Kędzior, 2016).

In recent years, there have been many substantial advances in active and passive remote sensing for the spatial mapping of soil moisture (Ustin & Middleton, 2021). However, these measurements are limited to surface soil moisture (SSM) (5–10 cm) (Li et al., 2023) and do not account for soil moisture in deeper layers (e.g., root zone soil moisture; RZSM), which is more critical for plant growth than SSM (Guo et al., 2023; Li et al., 2023). Therefore, algorithms were developed to accurately simulate the diffusion process of water and estimate a profile soil moisture, i.e., relating (remotely sensed) SSM and RZSM (Albergel et al., 2020; Ford et al., 2014; Li et al., 2023).

Numerous methods have been used to estimate RZSM from SSM, including data assimilation (Maggioni et al., 2013; Reichle et al., 2019), physical methods (Manfreda et al., 2014), neural networks (Grillakis et al., 2021), and deep learning algorithms (Babaeian et al., 2021). Data assimilation techniques are widely used to estimate RZSM at a large scale, which estimate RZSM by integrating the SSM observations into land surface models. The ensemble Kalman filter (EnKF) is one of the most widely used data assimilation techniques to estimate RZSM. The increasing availability of multiscale SSM datasets and improvement in EnKF methods over the years have strengthened this approach. However, EnKF is not only computationally expensive but also has limitations for nonlinear relationships between model states and observations (Clark et al., 2008; Yu et al., 2019). The most well-known application of data assimilation for RZSM is the soil moisture active and passive (SMAP) L4 product. It applies SMAP brightness temperature observations using EnKF to NASA’s land surface model (Reichle et al., 2019) providing global RZSM of 0–100 cm, at 9 km spatial resolution every 3 h. However, SMAP L4 aggregates soil moisture in the top 100 cm as RZSM and cannot directly provide RZSM dynamics at 0–30 cm which in turn mainly represents crop root layer for agricultural areas. Alternatively, reanalysis datasets, such as ERA5-Land (E5L) by the European Centre for Medium-Range Weather Forecasts (ECMWF), provide hourly RZSM at various depths with a spatial resolution of 10 km. Despite the high quality and decent resolution of E5L, the data becomes available only after a delay of 2 to 3 months, impeding its immediate application, e.g., in drought-related scenarios and for precision agriculture (Yang et al., 2022).

The exponential filter (EF) method proposed by Wagner (1998) transforms observed SSM series to dynamic signals representing soil moisture at deeper depths. Based on this transformation, the resulting soil water index (SWI) is linked to RZSM. The increase in optical and microwave satellite sensors can provide multisource SSM, facilitating near real-time RZSM simulations from the EF method. EF is simple and effective and requires only one key parameter, i.e., characteristic time length (*T*), and has been widely used to accurately simulate RZSM (Albergel et al., 2008; Ford et al., 2014; Pablos et al., 2018). The value of *T* reflects the combined effect of local conditions (soil and climatic variables) on the temporal persistence of soil moisture (Ceballos et al., 2005; Wang et al., 2017). To calculate SWI using the EF, an optimum *T* (*T*_opt_) is required, which is usually obtained through observed soil moisture at different depths. Under humid environmental conditions, a higher *T*_opt_ can generally be expected at greater soil depths. However, there is conflicting evidence regarding the relationship between *T*_opt_ and soil texture (Wang et al., 2017). Previous studies (Albergel et al., 2008; Ceballos et al., 2005; Ford et al., 2014) have also demonstrated that *T*_opt_ is affected by several climatic and environmental factors, so it is necessary to understand local controls on *T*_opt_.

Daily SWI data from Copernicus Global Land Services (CGLS) are produced by fusing SSM estimations from 25 km Metop ASCAT and Sentinel-1 sensors at a spatial resolution of 1 km over Europe (B Bauer-Marschallinger et al., 2018). This SWI product is generated from a recursive formulation of EF (Albergel et al., 2008) using a two-layer water balance model (Paulik et al., 2014) with eight fixed *T* lengths (2, 5, 10, 15, 20, 40, 60, and 100) and provided within 2 days of observation. Previous studies have shown the applicability of this product for hydrology, agricultural management, and ecosystem health (Fathololoumi et al., 2022). Madelon et al. (2023) evaluated several high and coarse-resolution datasets against in situ SSM observations over six regions and concluded that 1 km CGLS SWI and level-2 SMAP product (SMAP_L2_SM_SP) provided better estimates and temporal agreement than other high-resolution datasets. However, due to the strong spatial heterogeneity of soils and soil moisture, the continental information of SWI at 1 km resolution cannot be generalized at the local scale (Fathololoumi et al., 2022). Therefore, the depth specificity of CGLS SWI at finer resolution is of paramount importance for local RZSM estimations at different depths for precise agricultural monitoring.

Several downscaling methods have been proposed to obtain soil moisture with finer resolution. These methods include statistical downscaling (Chauhan et al., 2003; Piles et al., 2010), the disaggregation based on physical and theoretical changes in scale (Merlin et al., 2008), and machine learning (Im et al., 2016; Ke et al., 2016). The statistical and physical methods lack the ability to accurately describe the complicated relationships between soil moisture and auxiliary variables due to their inability to handle nonlinear relationships (Zhao et al., 2017). Machine learning methods (e.g., RF or neural network) have been widely used to obtain fine-resolution remote sensing products due to their ability to handle nonlinear relationships between auxiliary and prediction variables. Vegetation indices and the albedo, derived from remotely sensed optical/thermal data and topographical parameters, are good indicators for downscaling coarse-resolution soil moisture products (Peng et al., 2017). However, optical or thermal remote sensing data are limited to clear sky conditions due to their unavailability under cloud cover conditions. Several researchers have combined different synthetic aperture radar (SAR) data with optical data as input to machine learning algorithms for fine-resolution soil moisture mapping. SAR data is available in all-weather conditions and sensitive to surface water content (Bai et al., 2019). The normalized difference vegetation index (NDVI) and SAR backscatter have been combined for SSM mapping using support vector regression (SVR) (Holtgrave et al., 2018) and artificial neural network (ANN) (El Hajj et al., 2019). Downscaling methods based on active SAR sensors such as Sentinel-1 usually leveraged linear or nonlinear relationship between SAR backscatter and soil moisture data in time series. However, backscatter from active SAR sensors is greatly influenced by surface roughness and vegetation limiting their applications in most areas (Reuß et al., 2024). Nonetheless, these are considered time-invariant for longer time series (He et al., 2018).

Previous studies have largely focused on the use of vegetation indices and topographic and thermal characteristics to establish the relationship with soil moisture (Fathololoumi et al., 2020; Lv et al., 2021; Montzka et al., 2018; Peng et al., 2017). In addition, these studies have estimated soil moisture with greater than 500 m spatial resolution, but local applications require fine resolutions. There is only one previous study on the downscaling of CGLS SWI beyond 100 m resolution available (Fathololoumi et al., 2022). They utilized optical images, environmental, and topographical parameters together with RF for downscaling and focused on soil moisture at 5 cm depth. To our knowledge, so far, no study has utilized the combined capability of SAR and optical data to downscale 1 km CGLS SWI to a resolution of < 500 m for assessing RZSM on cropland.

In order to fill this gap and to achieve local scale estimates of RZSM in regular intervals, we propose a downscaling procedure for CGLS 1 km SWI to 100 m resolution at the example of an intensively used agricultural landscape in Mecklenburg-Western Pomerania, Germany (DEMMIN). The objectives of this study are as follows: (1) to calibrate 1 km CGLS SWI datasets against observed RZSM at depths ranging from 10 to 60 cm for selecting the depth-wise *T*_opt_, (2) to conduct a comparative analysis to investigate the effect of SAR and optical features in downscaling the CGLS SWI at 100 m spatial resolution using RF, and (3) to evaluate the downscaled SWI against observed RZSM. For independent validation of *T*_opt_, E5L data was utilized. This study investigates the use of various input dataset combinations for RF-based downscaling of the 1 km CGLS SWI dataset and the estimation of high-resolution RZSM at different depths in agricultural landscapes under temperate climate conditions for the first time.

## Material and methods

### Study area

**Fig. 1:**
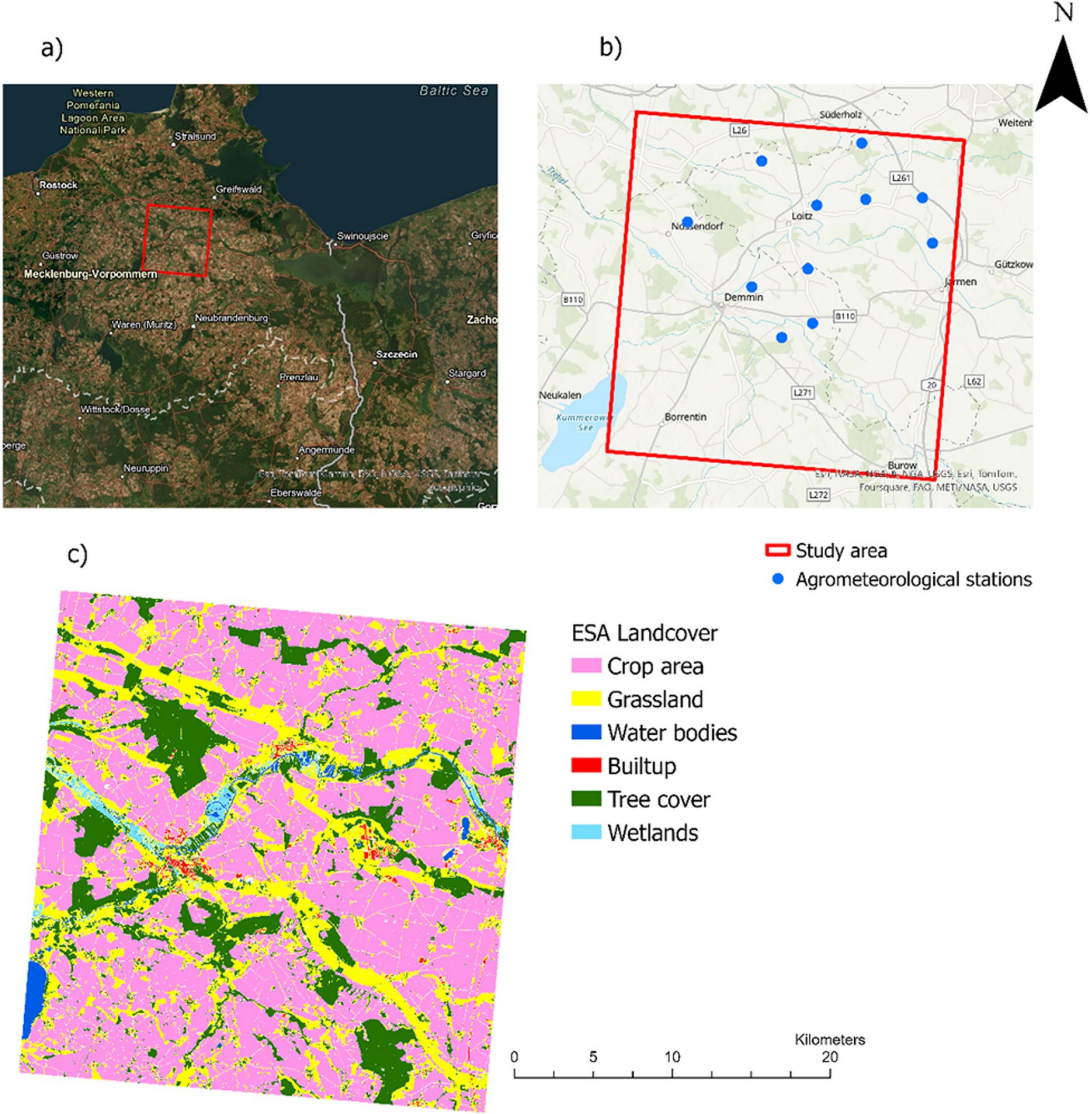
** a** Location of the study area, **b** location of agrometeorological stations used in the study, **c** land cover map developed by European Space Agency (ESA) (Zanaga et al., 2021 ).

The study was carried out in the DEMMIN study area in Mecklenburg-Western Pomerania, Germany (Fig. 1). The climate conditions in the study area are characterized by an average air temperature of 8.3 °C and an annual precipitation of about 550 mm, classifying it as temperate Middle-European climate with perennial humidity (Borg, 2009). Out of the 1.34 million hectares of agricultural area in Mecklenburg-West Pomerania, which is about 57% of the total area, about 80% are used as cropland, while the remaining 20% are permanent grassland (Heupel et al., 2018). The dominated soils found in this region are mainly loamy sands and sandy loams (Hosseini et al., 2021).

Since 2001, DEMMIN has served as a calibration and validation test site for earth observation missions conducted by the German Aerospace Center (DLR). In Germany, the network of Terrestrial Environmental Observatories (TERENO) was established in 2008 to facilitate long-term environmental research. DEMMIN experimental site has been an integral part of the TERENO network since 2011, specifically contributing to agricultural research and the integration of remote sensing data with in situ measurements (Zacharias et al., 2011). With the same aims, DEMMIN contributes to the Joint Experiment for Crop Assessment and Monitoring (JECAM) by NASA (e.g., Hosseini et al. (2021)).

### Ground data

From the TERENO network at DEMMIN (https://www.tereno.net/) (Itzerott et al., 2018), 11 agrometeorological stations (Fig. 1) equipped with soil moisture sensors placed at 10 cm intervals from 10 to 100 cm below the surface were selected for calibration and validation.

**Fig. 2:**
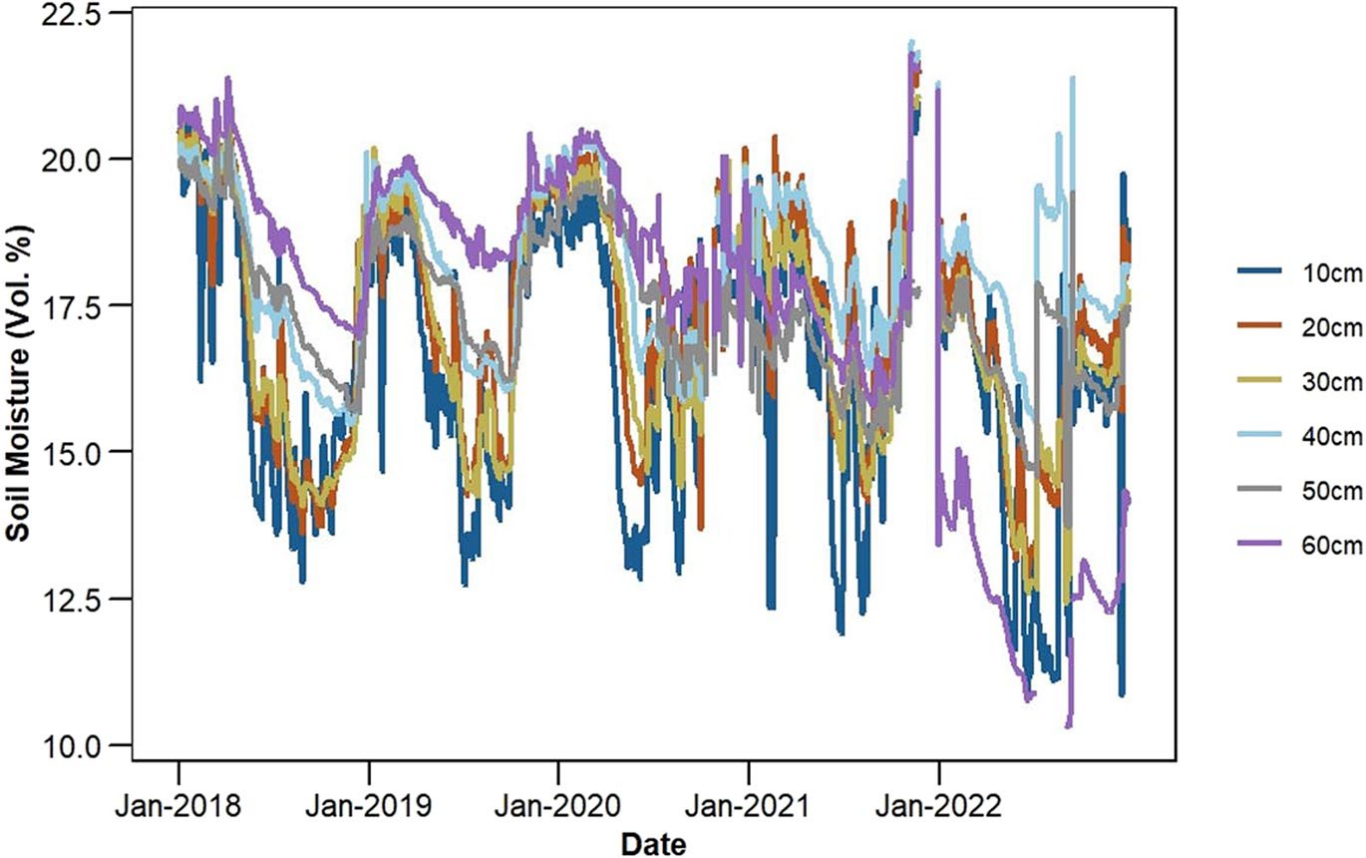
Depth-distributed mean soil moisture from 2018 to 2022, averaged across all 11 agrometeorological stations used in this study

To account for RZSM, we collected soil moisture data within the depth range of 10 to 60 cm, recorded at 10 cm intervals available with the agrometeorological stations (Fig. 1) from 2018 to 2022. The data is available at 15-min intervals and was subsequently averaged to a daily scale. In general, the amount of data decreased with the increase in root zone depth. The highest and lowest data availability is at 20 cm and 60 cm depths, respectively. In terms of year-wise data availability, the year 2018 shows the highest count across all depths. Figure 2 presents the average RZSM at each depth across all stations from 2018 to 2022. The figure illustrates the variations in soil moisture content at different soil depths. The standard deviation (SD) of soil moisture across depths from 10 to 50 cm ranges from 1.35 to 2.53% (Vol.%). The highest SD was observed at 10 cm, while the lowest SD occurred at 50 cm. This trend indicates a decrease in soil moisture variability with increasing depth from 10 to 50 cm. However, at 60 cm, the soil moisture variability increased again with SD of 2.38 (Vol.%), showing more variability than the depths from 20 to 50 cm.

### Datasets

#### Soil water index (SWI)

Daily 1 km SWI data with eight fixed *T* (2, 5, 10, 15, 20, 40, 60, 100) (collectively referred as SWI_T-1 km_) is derived from the fusion of SSM observations from Sentinel-1 C-band SAR and Metop ASCAT sensors called SCATSAR-SWI, described in detail in Bauer-Marschallinger et al., (2018, 2020). The data is available from the CGLS website (https://land.copernicus.eu/) since January 2015.

SWI attempts to estimate RZSM from observed SSM using EF proposed by Wagner et al. (1999). The estimation of SWI is based on a two-layer water balance model, with topsoil representing the first layer (~ 5 cm) and the second layer extending from the bottom of the first layer. It assumes RZSM is linked to SSM, and any wetting and drying in the surface influences the RZSM. The recursive formulation of EF by Albergel et al. (2008) was used to derive SWI_T-1 km_ datasets and gives as

1 $${SWI}_{tn}={SWI }_{tn-1}+{K}_{tn} ({ms }_{tn}-{SWI }_{tn-1})$$

where *t*_n_ and *t*_n-1_ are the observation time of current and previous normalized SSM (ms) measurements in Julian days, respectively. SWI_tn_ and SWI_tn-1_ are estimated RZSM at time *t*_n_ and *t*_n-1_, respectively, and *K*_tn_ is gained at time *t*_n_ and is given as

2 $${K}_{tn}=\frac{{K }_{tn-1}}{{K t}_{n-1}+ {e}^{-(\frac{tn{- t}_{n-1}}{T})}}$$

The formulation is initiated using *K*_0_ = 1, SWI_0_ = ms_(t0)_. The parameter *T* is an empirical parameter, and it represents a characteristic time length (referred to in days). It regulates the degree of smoothing in the SSM series and determines the response time to changes in the surface wetness conditions.

We obtained daily CGLS SWI_T-1 km_ data from 2018 to 2022 along with a surface state flag (SSF) to remove the SSM measurement made under freezing conditions. This is due to the decrease in the radar backscatter signal under frozen conditions, resulting in unrealistic SSM values (Wagner, 1998).

#### SAR data

Sentinel-1 is a part of the ESA Copernicus program, which consists of two satellites, A and B. Both satellites operate in opposite sun-synchronous orbits at an altitude of 693 km and carry a C-band active SAR sensor (5.405 GHz). They offer reliable observations in all weather conditions. We acquired C-band Sentinel-1 level-1 ground range detection (GRD) images in dual polarization (VV and VH) in interferometric wide swath (IW) acquisition mode from Google Earth Engine (GEE) (https://earthengine.google.com/). We used the GEE implementation provided by Mullissa et al. (2021) to apply additional processing steps (speckle filtering), which are not available on the ingested Sentinel-1 imagery. Both satellites A and B have a similar orbit configuration. By using data from both satellites, the temporal resolution was increased from 12 to 6 days in a single pass (ascending or descending). This study also jointly used the observations from ascending and descending passes, which further increased the temporal resolution to ~ 3 days. The backscatter coefficient (*σ*^0^) data for both VV (*σ*_vv_^0^) and VH (*σ*_vH_^0^) polarizations were obtained during both ascending and descending passes in 2018. The spatial resolution was originally 10 × 10 m but was resampled to 100 m using the nearest neighbor technique. We obtained *σ*_vH_^0^and *σ*_vv_^0^ from 121 Sentinel-1 (A & B) acquisitions in both ascending and descending passes over the study area during 2018.

In addition to the *σ*_vv_^0^ and *σ*_vH_^0^, we also used calculated radar vegetation index (RVI). Kim and Van Zyl (2009) and Trudel et al. (2012) developed the radar vegetation index (RVI) for quad-polarized SAR data. Trudel et al. (2012) later adapted this index for dual-polarized SAR data, assuming that *σ*_HH_^0^≈*σ*_vv_^0^ and *σ*_Hv_^0^≈*σ*_HH_^0^. Kim et al. (2012) have reported that RVI is less sensitive to environmental changes making it useful for vegetation monitoring using SAR data. Recently, Kim and van Zyl (2004) proposed an adaptation of the modified index for Sentinel-1 (S1) data, given in Eq. 3.

3 $$RVI=\frac{4\times {\sigma }_{vH}^{0}}{{\sigma }_{vH}^{0}+{\sigma }_{vv}^{0}}$$

#### Optical data

Sentinel-2 Multispectral Imager (MSI) Level-2A surface reflectance data was obtained from GEE. Sentinel-2 mission comprises of two identical optical satellites: Sentinel-2A and Sentinel-2B. The launch of both satellites Sentinel-2 A and B in 2015 and 2017, respectively, helped to half the revisit time of the Sentinel-2 mission from 10 to 5 days. The Sentinel-2 provides multispectral data in 13 bands with a spatial resolution of 10, 20, and 60 m. Out of these 13 bands, bands 1, 9, and 10 are dedicated for atmospheric correction and cloud screening. The optical data is challenged by cloud cover and cloud shadows, which affect its spatial coverage. For 2018, a total of 30 Sentinel-2 acquisitions with at least 50% of cloud-free pixels were collected. Cloudy pixels were subsequently masked out using the cloud coverage band (QA60).

Several optical indices are used extensively for soil moisture retrieval (Hegazi et al., 2023). The most common are normalized difference vegetation index (NDVI) (Merzlyak et al., 1999), normalized difference water index (NDWI) (Gao, 1996), global vegetation moisture index (GVMI) (Ceccato et al., 2002), and fraction vegetation cover (FVC) (Wakigari & Leconte, 2022). These indices were calculated using expressions 4 to 7. These indices were resampled to 100 m using the nearest neighbor technique.

4 $$NDVI=\frac{NIR- RED}{NIR+RED}$$

5 $$NDWI=\frac{NIR- SWIR 1}{NIR+SWIR 2}$$

6 $$GVMI=\frac{(NIR+0.1)-(SWIR+0.02)}{\left(NIR+0.1\right)+(SWIR+0.02)}$$

RED (band 4), NIR (band 8), SWIR 1 (band 11), and SWIR 2 (band 12) were used to calculate these indices.

FVC is typically calculated from NDVI (Carlson & Ripley, 1997; Ermida et al., 2020). We used the relationship provided by Carlson and Ripley (1997) to calculate the FVC given in 7.

7 $$FVC={\left(\frac{NDVI- ND{VI}_{Bare}}{ ND{VI}_{Veg}- ND{VI}_{Bare}}\right)}^{2}$$

where NDVI_Bare_ and NDVI_Veg_ correspond to the NDVI of completely bare and fully vegetated pixels, respectively. Previous studies have established NDVI_Bare_ and NDVI_Veg_ values of 0.18 and 0.85, respectively. However, some studies apply NDVI_Veg_ = 0.5; Jiménez-Muñoz et al. (2009) showed that for high-resolution data, NDVI_Veg_ ranges from 0.8 to 0.9. Pixels with values below 0.18 are considered completely bare, while those above 0.85 are considered fully vegetated.

#### Topographical parameters

Surface topography is one of the most important variables affecting SWI. It serves as the primary factor influencing the spatial variation of hydrological conditions, thereby controlling the spatial distribution of SWI. The flow of groundwater often aligns with the contours of surface topography, making topographic parameters essential for examining SWI spatial patterns (Raduła et al., 2018). In this study, we used elevation, slope, aspect, and topographical wetness index (TWI) as topographic indices. The correlation between elevation and SWI is direct, as highlighted by Firozjaei et al. (2020). Also, higher slopes contribute to higher soil water content and vice versa (Magdić et al., 2022). Similarly, the aspect has a certain influence on the distribution of soil moisture and is also closely related to surface topography and vegetation cover (Chen et al., 2019).

TWI is used in hydrological analysis to describe an area’s tendency to accumulate water. It quantifies the influence of topography on runoff production (Fathololoumi et al., 2022). TWI was calculated using a specific catchment area (SCA) and slope angle (*φ*) as follows:

8 $$TWI= \text{ln}\left(\frac{\text{SCA}}{\text{tan} \phi}\right)$$

We obtained the 1 arc second (~ 30 m) Digital Elevation Model (DEM) of the Shuttle Radar Topography Mission (SRTM) from GEE and subsequently calculated the slope, aspect, and TWI using System for Automated Geoscientific Analyses (SAGA) platform (Conrad et al., 2015). Afterwards, these indices were resampled to 100 m using the nearest neighbor technique to match the spatial resolution with other datasets.

#### ERA5-Land reanalysis data

ERA5-Land (E5L) serves as a dataset that specifically focuses on the land component of the ERA5 climate reanalysis. This dataset was obtained through the downscaled ERA5 reanalysis data-driven ECMWF land surface model TESSEL and was made accessible by ECMWF (Wu et al., 2021). Covering the period from 1981 to the present, the E5L offers important environmental variables (available at https://cds.climate.copernicus.eu/cdsapp#!/home). E5L soil moisture dataset offers a comprehensive four-layer soil moisture dataset (Layer 1, 0 to 7 cm; Layer 2, 7 to 28 cm; Layer 3, 28 to 100 cm; Layer 4, 100 to 268 cm), characterized by high spatial and temporal resolution (0.1° and 1 h).

Due to the focus of this study in the root zone depth range from 10 to 60 cm, we obtained hourly E5L soil moisture data for Layer 2 and Layer 3, covering the period from 2018 to 2022. Subsequently, we aggregated the hourly data to calculate the daily average soil moisture for Layers 2 and 3. This E5L RZSM was utilized to validate the *T*_opt_ calibration of SWI_T-1 km_ conducted against in situ data.

## Methodology

### Calibration of time length

Before starting the downscaling process of SWI_T-1 km_ for high-resolution RZSM at different depths, the first step is to calibrate the SWI_T-1 km_ dataset with eight *T* values to obtain *T*_opt_ for each depth of the study area. The calibration of *T* was performed using in situ RZSM data available at depths ranging from 10 to 60 cm, with a 10 cm interval against SWI_T-1 km_. The selection of *T*_opt_ requires long-term SWI_T-1 km_ and observed RZSM observations. Therefore, we used the observed RZSM and SWI_T-1 km_ dataset at the agrometeorological stations from 2018 to 2022. The selection of in situ data at each depth follows the criterion that at least 100 concurrent daily values must be available for both the observed RZSM and SWI_T-1 km_ time series. Monthly aggregates were used to minimize the impact of outliers in the daily data of both the observed and SWI_T-1 km_ datasets (Grillakis et al., 2021). Pearson’s correlation coefficient (*R*) was then calculated by comparing the depth-wise observed RZSM with SWI_T-1 km_ for each station. The depth-wise *T*_opt_ of each station was then determined based on the highest *R* obtained. Subsequently, the overall *T*_opt_ for each depth was selected based on the mode value of *T*_opt_ from all stations in the study area. Further validation of *T*_opt_ was done by repeating the same methodology using E5L Layers 2 and 3 RZSM against SWI_T-1 km_ at agrometeorological sites.

The SWI is a relative soil moisture given in percentage ranges between 0 (dry) and 100 (wet), while in situ measurements are expressed in the volumetric units (Vol.%). For meaningful comparison between SWI and in situ RZSM, the SWI is converted to SWI^*^, to have the same mean and standard deviation of ground observations (Vol.%). Various methods are available for the rescaling of SWI to SWI^*^, such as linear regression (Jackson et al., 2010), linear transformation (Brocca et al., 2010), and cumulative density function (CDF) matching (Brocca et al., 2011). However, none of these methods significantly alters the correlation coefficient (Paulik et al., 2014). We employed linear transformation using Eq. 9.

9 $${SWI}_{T}\times \left(t\right)=\frac{{SWI}_{T}\left(t\right)- \overline{{SWI }_{T}}}{\text{SD }\left({SWI}_{T}\right)}\times SD (SM)+ \overline{SM }$$

where $\overline{SM}$ and SD (SM) are the mean and standard deviation of ground soil moisture observations, respectively. Similarly, $\overline{{SWI }_{T}}$ and SD (SWI_T_) are the mean and standard deviation of SWI_T-1 km_, respectively.

### Random forest–based downscaling

RF is a machine learning method that can be used for both classification and regression tasks (Breiman et al., 1984). It creates an ensemble of decision trees, where in each tree, a random subset of the features is selected for splitting at each node, and the best split is chosen based on a certain criterion (e.g., Gini impurity). Using a high number of decision trees can reduce the generalization error and help overcome issues of overfitting due to correlated variables (Liaw & Wiener, 2002). The predictions of the trees are then combined, usually by taking the mean or mode of the individual tree predictions, to produce the final estimate. RF is a widely used and convenient machine learning algorithm with a high accuracy for downscaling purposes as previously shown by Liu et al. (2020). The spatial downscaling method is based on the relationship between SWI_T-1 km_ and surface and environmental variables as detailed in Table 1.

**Table 1:** List of optical, SAR, and topographical variables utilized for downscaling

Datasets	Variable name	Code	Software/platform
Optical	Normalized difference vegetation index	NDVI	GEE
	Normalized difference water index	NDWI	GEE
	Global vegetation moisture index	GVMI	GEE
	Fraction vegetation cover	FVC	GEE
SAR	VV backscatter coefficient	VV	GEE
	VH backscatter coefficient	VH	GEE
	Radar vegetation index	RVI	GEE
Topographical	Elevation	Elevation	SRTM
	Slope	Slope	SAGA
	Aspect	Aspect	SAGA
	Topographical wetness index	TWI	SAGA

The relationship between SWI_T-1 km_ and surface and environmental features (Table 1) at coarser resolutions is established. Subsequently, this relationship is applied to higher-resolution surface and environmental features data. The downscaling was undertaken for the SWI_T-1 km_ based on *T*_opt_ results (Table 2). The downscaling process was performed for the year 2018 because of the highest availability of ground observations.

**Table 2:** Selected *T*_opt_ for each soil depth

**Depth (cm)**	10	20	30	40	50	60
*T*_opt_ (days)	20	40	60	100	100	100

The specific steps for downscaling the SWI used in this study are as follows:

1. The surface and environmental parameters were resampled to 1 km to match the spatial resolution of SWI_T-1 km_ after masking out areas other than crop and grassland using the ESA land cover map.
2. We randomly split the dataset, with 70% for the training and the remaining 30% for the validation of the model.
3. The model developed in step 2 was applied to high-resolution (100 m) auxiliary parameters to predict the high-resolution PreSWI_T-100 m_.
4. The improvement in spatial distribution of RF downscaling after residual correction is common in precipitation and soil moisture downscaling (Tang et al., 2021). Residual correction is also a necessary step for correcting the prediction error in data-driven downscaling methods (Zhu et al., 2023). Hence, the PreSWI_T-100 m_ (step 3) was resampled to 1 km and subtracted from the original SWI_T-1 km_ to calculate residuals (Residual_-1 km_).
5. The Residual-_1 km_ was resampled to 100 m and added to PreSWI_T-100 m_, and the final SWI_T-100 m_ was estimated at 100 m resolution.
6. The final residual corrected SWI_T-100 m_ map was converted to SWI^*^_T-100 m_ (Vol.%) and evaluated against observed RZSM measured at different depths.

**Fig. 3:**
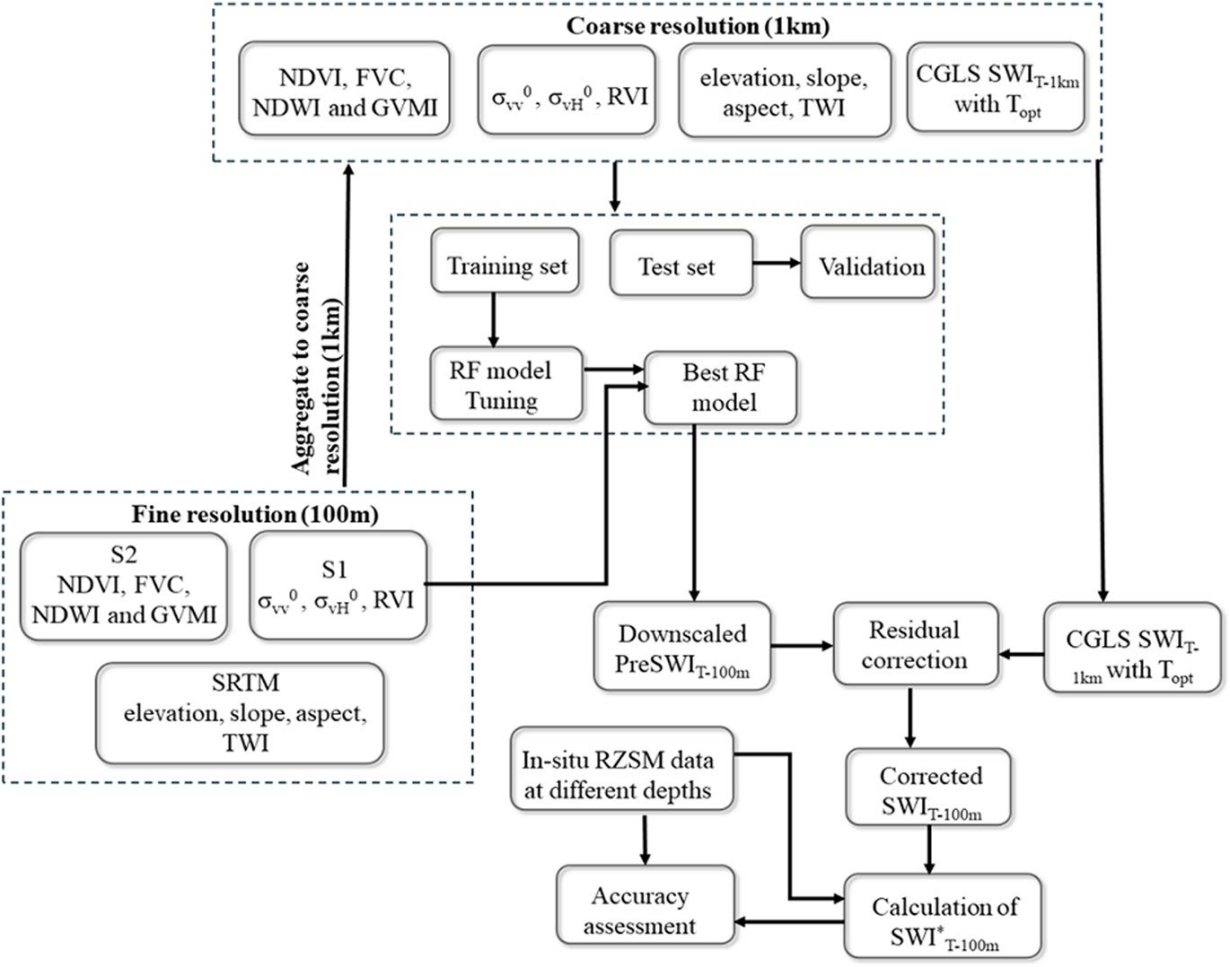
Workflow adopted for downscaling of SWI_T-1 km_. SWI_T-1 km_, 1 km SWI dataset; RF, random forest; S1, Sentinel-1; S2, Sentinel-2; FVC, fractional vegetation cover; NDVI, normalized difference vegetation index; NDWI, normalized difference water index; GVMI, global vegetation moisture index; RVI, radar vegetation index; σ_vv_^0^, backscatter coefficient in VV polarization; σ_vH_^0^, backscatter coefficient in VH polarization; SRTM, Shuttle Radar Topographic Mission; TWI, topographical wetness index; PreSWI_T-100 m_, predicted SWI at 100 m before residual correction; SWI_T-100 m_, downscale SWI at 100 m resolution after residual correction; RZSM, root zone soil moisture; SWI^*^_T-100 m_, SWI_T-100 m_ converted to Vol.%

The flowchart of the downscaling methods is presented in Fig. 3.

The use of SAR data in the downscaling process would increase the applicability due to cloud independence, and it can effectively address gaps in optical coverage. In addition, the effect of the remotely sensed variables, i.e., Sentinel-based features, on the downscaling of SWI_T-1 km_ with respect to *T*_opt_ could be significant, and the spatial distribution of downscaled SWI_T-100 m_ can also provide additional insight into the usefulness of these optical and SAR variables in downscaling. Therefore, three sets of RF models for comparative analysis were established. RF1 vs RF2 and RF1 vs RF3 and RF2 vs RF3 were used to show the effects of the SAR and optical variables independently and together. These RF models are given as follows:

SWI_T_ = RF1 (*σ*_vv_^0^, *σ*_vH_^0^, RVI, elevation, slope, aspect, TWI).

SWI_T_ = RF2 (NDVI, NDWI, GVMI, FVC, elevation, slope, aspect, TWI).

SWI_T_ = RF3 (*σ*_vv_^0^, *σ*_vH_^0^, RVI, NDVI, FVC, NDVI, NDWI, GVMI, FVC, elevation, slope, aspect, TWI).

The RF algorithm includes the variable importance function to evaluate the contribution of each variable to the model’s performance. This is achieved by using out-of-bag (OOB) samples, where the value of each variable is randomly permuted, while others remain unchanged. The resulting prediction error (mean square error (MSE) for regression) across all trees is averaged to determine the importance of each variable (Liaw & Wiener, 2002). Importance is measured by the percentage increase in the MSE when a variable is permuted, indicating its impact on the accuracy of the model. In general, higher MSE values indicate a higher importance of the predictor, enhancing the prediction accuracy of the RF model.

### Evaluation metrics

Two evaluation metrics were used, i.e., the correlation coefficient (*R*) (Eq. 10) and the root mean square error (RMSE) (Eq. 11). Firstly, we used *R* between converted SWI^*^_T-1 km_ (Vol.%) and observed RZSM for calibration of *T*. Secondly, we evaluated the performance of each RF model on the test set and high-resolution predictions (SWI_T-100 m_) against SWI_T-1 km_. Finally, the converted SWI^*^_T-1 km_ and SWI^*^_T-100 m_ datasets were compared against observed RZSM from ground stations.

10 $$\text{R}=\frac{{\sum}_{i=1}^{n}\left({P}_{i}^{o}+\overline{{P }_{i}^{o}}\right)\left({P}_{i}^{e}+\overline{{P }_{i}^{e}}\right)}{{\sum}_{i=1}^{n}{\left({P}_{i}^{o}+\overline{{P}_{i}^{o}}\right)}^{2}{\left({P}_{i}^{e}+\overline{{P}_{i}^{e}}\right)}^{2}}$$

11 $$RMSE=\sqrt{\frac1n {\sum}_{i=1}^{n}(P_i^e-P_i^o )^2}$$

*P*_i_^e^ and *P*_i_^o^ represent the estimated and measured values of the *i*-th sample, respectively. $\overline{{P}_{i}^{o}}$ and $\overline{{P }_{i}^{e}}$ represent the mean of the measured and estimated time series values in the comparison.

## Results

### Depth-wise optimum time length

**Fig. 4:**
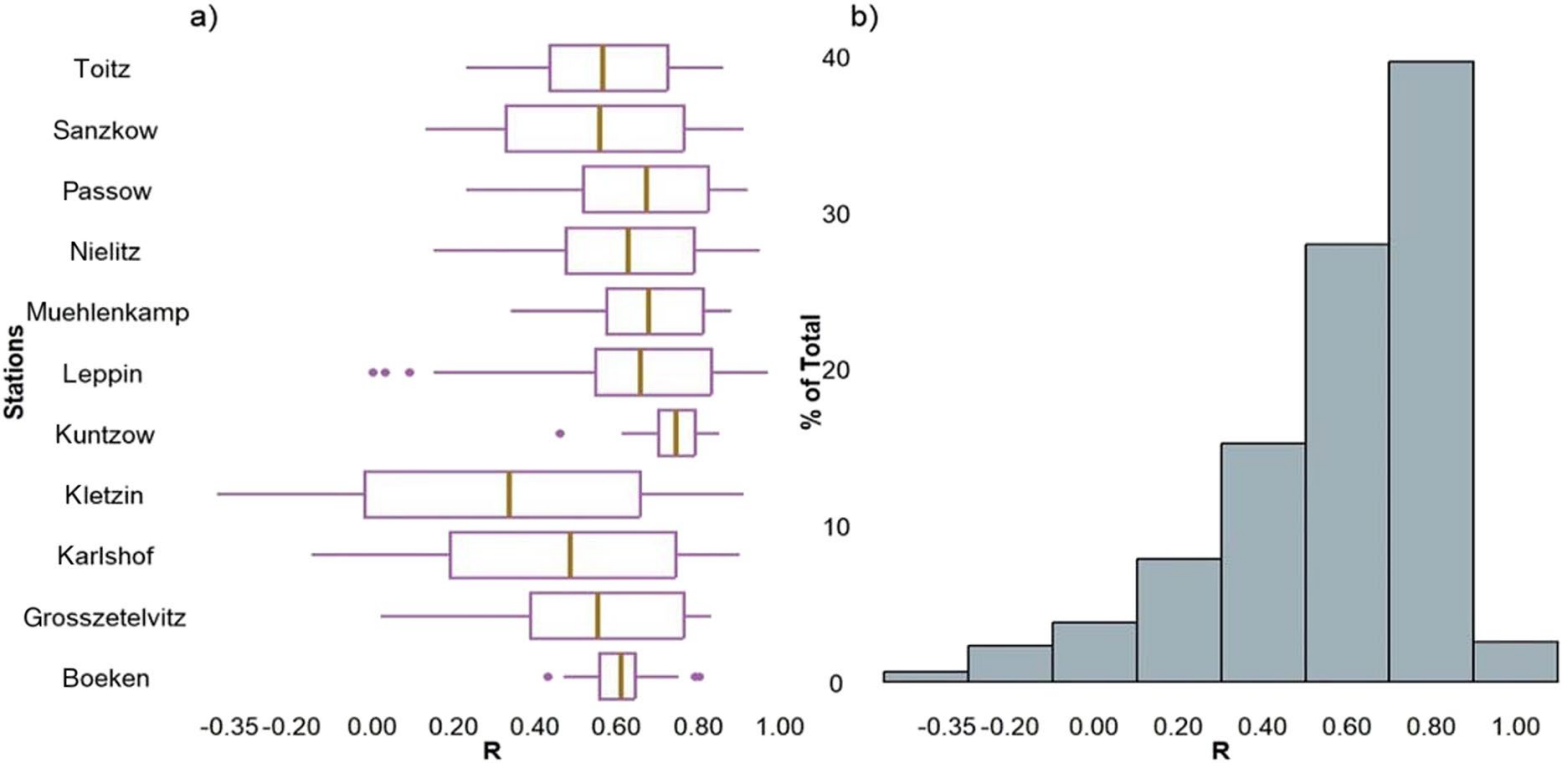
**a** Box plots showing station-wise correlation coefficient between SWI^*^_T-1 km_ and depth-wise observed RZSM data. **b** Histogram distribution of correlation coefficients presented in Fig. 4a. R , Pearson’s correlation coefficient; SWI^*^_T-1 km_, converted 1 km SWI dataset; RZSM, root zone soil moisture.

The average *R* between SWI^*^_T-1 km_ and available observed RZSM at different depths in the study area is 0.59 and is greater than 0.5 for 70% of the time series (Fig. 4).

**Fig. 5:**
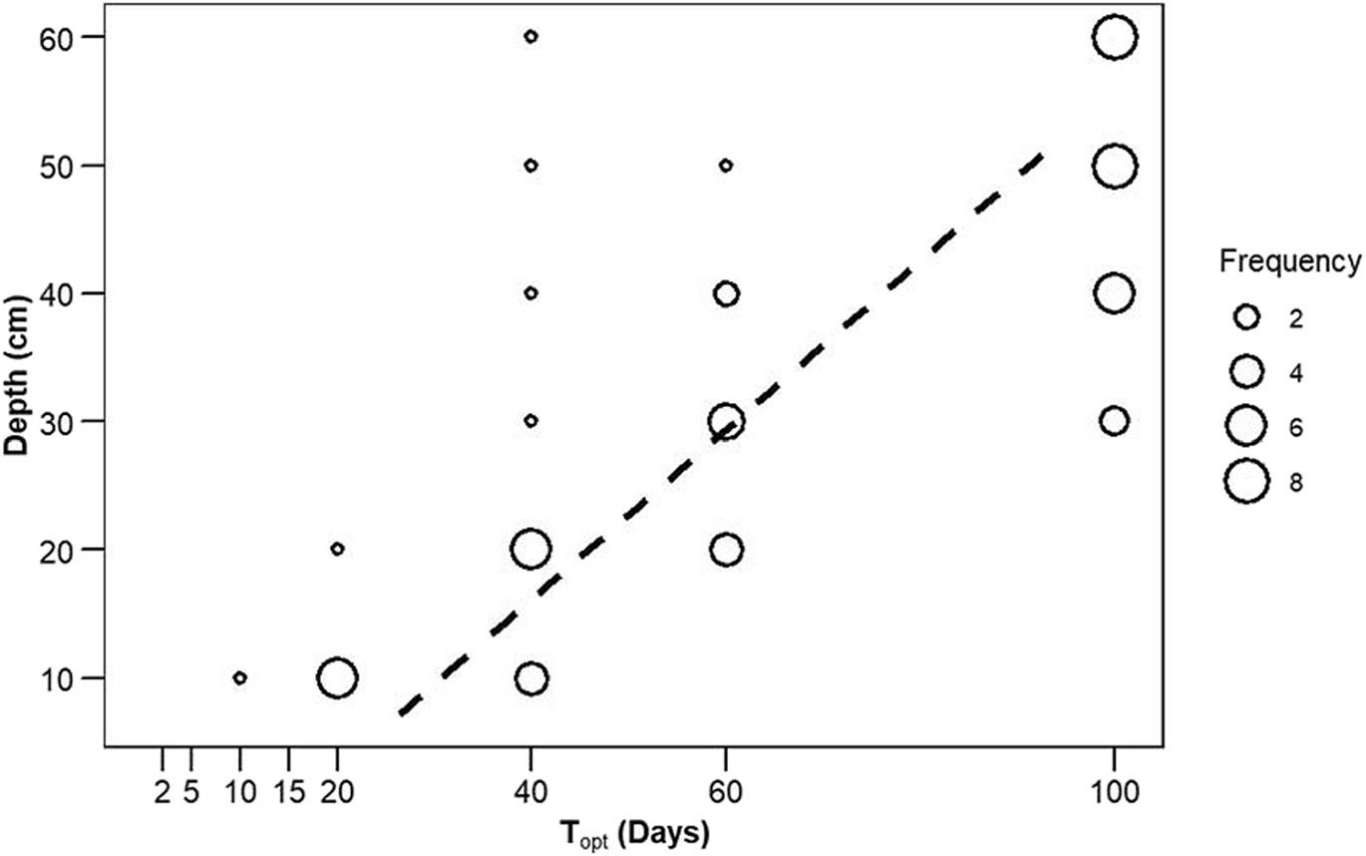
*T*_opt_ against soil depth. The point size reflects the number of stations (frequency) resulting in the same *T*_opt_. The dotted line shows the linear model fitted between average *T*_opt_ and soil depth. *T*_opt_, optimized time length

The *T*_opt_ increases with soil depth (Fig. 5). A significant positive relationship was observed between average *T*_opt_ and average soil depth with a coefficient of determination (*R*^2^) equal to 0.61 (*p*-value = 2.952e − 13). The specific selected *T*_opt_ for each depth is provided in Table 2, derived from the depth-wise frequency of *T*_opt_, as presented in Fig. 5.

*T*_*opt*_ optimized time length (20, 40, 60, 100).

As the depth of the soil increases, the *T*_opt_ value exhibits an increasing trend, notably from 10 to 30 cm. Additionally, within 40–60 cm, *T*_opt_ consistently resulted in 100. This observation indicates a decreasing variability in soil moisture with an increase in soil depth (Albergel et al., 2013).

To further validate the selected *T*_opt_ for each depth, we applied daily Layers 2 and 3 RZSM from E5L. Employing the same methodology, we compared E5L and SWI_T-1 km_ datasets at agrometeorological locations. All stations resulted in *T*_opt_ = 40 days for Layer 2 and *T*_opt_ = 100 days for Layer 3, indicating high consistency of *T*_opt_ derived from E5L observation data (Table 2).

### Performance of different downscaling models

Table 3 shows the *R* and RMSE on test sets of RF models for downscaling of selected SWI_T-1 km_ (*T*_opt_, 20, 40, 60, 100). Over the range of *T*_opt_ (20 to 100 days), RF3 consistently outperformed RF1 and RF2 in terms of accuracy. Among the RF models, RF1 which used SAR and topographical variables, *R* decreased from 0.61 to 0.50 as *T*_opt_ increased from 20 to 100 days. Contrarily, RF2 established using optical instead of SAR variables exhibited an increase in *R* from 0.65 (*T*_opt_ = 20) to 0.67 (*T*_opt_ = 100). RF2 consistently outperformed RF1, and the correlation coefficient difference between RF1 and RF2 increased with increasing *T*_opt_. The combined use of SAR and optical data (RF3) produces better results than RF1 and RF2, also with a declining trend from 0.84 (*T*_opt_ = 20) to 0.79 (*T*_opt_ = 100). However, this decrease in *R* with RF3 is not as pronounced as with RF1, possibly due to the addition of optical variables.

**Table 3:** Performance of RF models on test sets for downscaling SWI_T-1 km_ with selected *T*_opt_ (Table 2)

**T**_**opt**_ **(days)**	RF models	*R*	RMSE (%)
20	**RF1**	0.61	12.21
	**RF2**	0.65	11.20
	**RF3**	0.84	8.40
40	**RF1**	0.54	12.45
	**RF2**	0.67	9.80
	**RF3**	0.82	8.33
60	**RF1**	0.51	11.80
	**RF2**	0.67	9.90
	**RF3**	0.81	7.97
100	**RF1**	0.50	9.70
	**RF2**	0.67	9.10
	**RF3**	0.79	6.60

*RF* random forest, *SWI*_*T-1 km*_ 1 km SWI dataset, *T*_*opt*_ optimized time length.

Analogously, RF3 revealed the lowest RMSE. The decrease in RMSE with increased *T*_opt_ is related to the decrease of variability and smoother time series of soil moisture with an increase in the depth of the soil.

### Feature importance

**Fig. 6:**
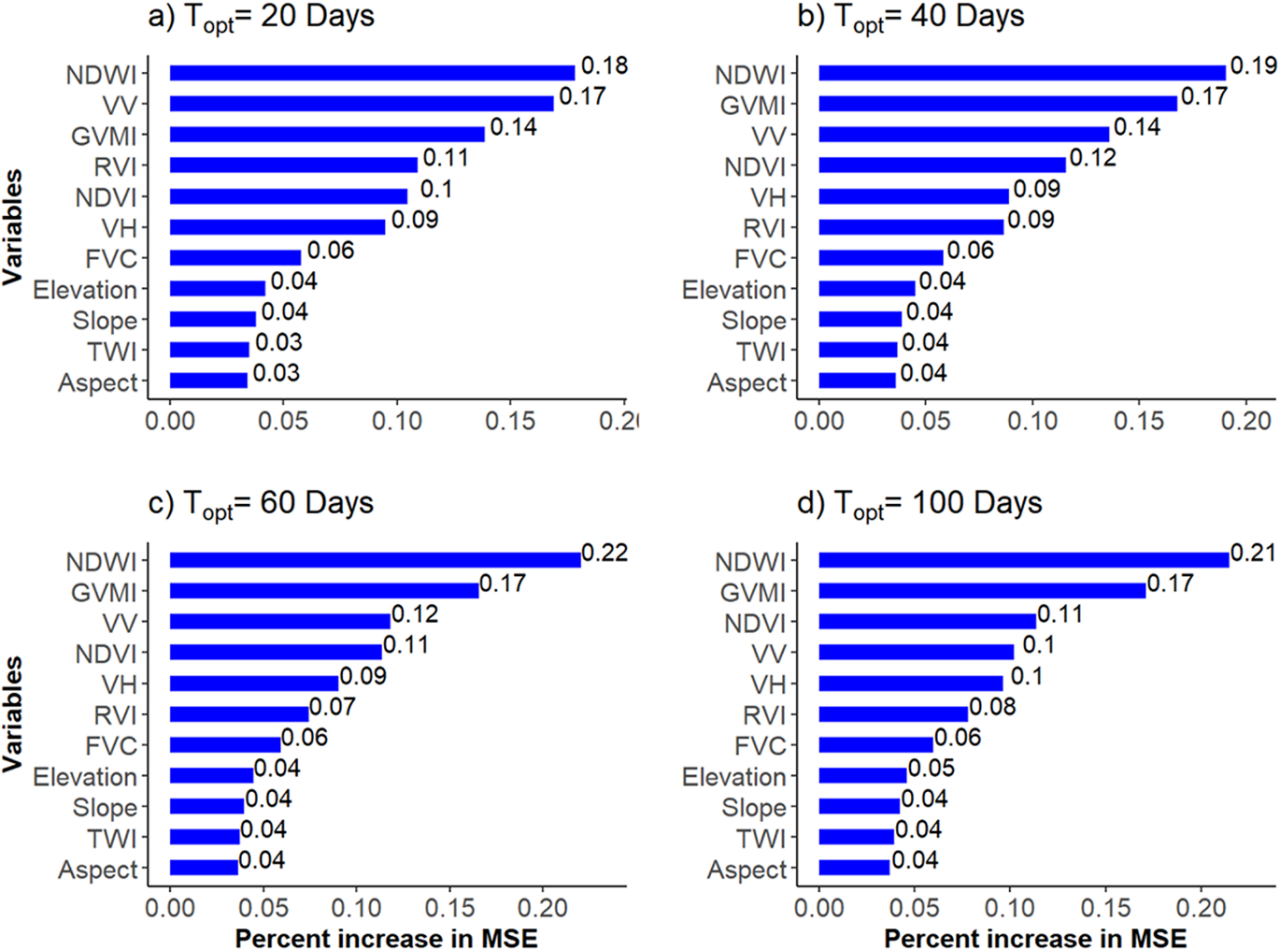
Importance of variables used in RF3 model for downscaling of SWI_T-1 km_ based on *T*_opt_**a** 20 days, **b** 40 days, **c** 60 days, and **d** 100 days. RF, random forest; SWI_T-1 km_, 1 km SWI dataset; *T*_opt_, optimized time length; MSE, mean square error

The NDWI exhibited the highest importance among variables (Fig. 6). This consistently persisted across the *T*_opt_ values; NDWI’s importance increased slightly from 0.18 to 0.21 with an increase in *T*_opt_ from 20 to 100 days. Among the SAR variables used in this study, *σ*_vv_^0^ had the highest importance, ranking as the second most important variable after NDWI with *T*_opt_ = 20 days. However, its importance declined from 0.17 to 0.1 as *T*_opt_ increase to 100 days. The decreasing importance of *σ*_vv_^0^ with increasing is linked to soil layer depth, while the importance of *σ*_vH_^0^ remains consistent. This figure also shows that the importance of optical variables, excluding the FVC, increased with the increase of *T*_opt_, whereas the importance of SAR features decreased. This decline in the importance of SAR variables is also reflected in the performance of the RF models (Table 3). Specifically, as the importance of SAR variables decreased with increasing *T*_opt_, the accuracy trend of RF1 models follows a similar pattern with the decline in *R*. In contrast, the *R* achieved using RF2 increased slightly from 0.65 to 0.67.

Additionally, from optical variables, NDWI and GVMI were found to be more important than NDVI. RVI and FVC had consistent but lower importance compared to other optical and SAR variables. Moreover, the importance of topographical parameters used were approximately similar and were not significant contributors to the performance of the RF models, which is likely due to the lower elevation gradient in DEMMIN.

### Spatial distribution and comparison of downscaled SWI

**Fig. 7:**
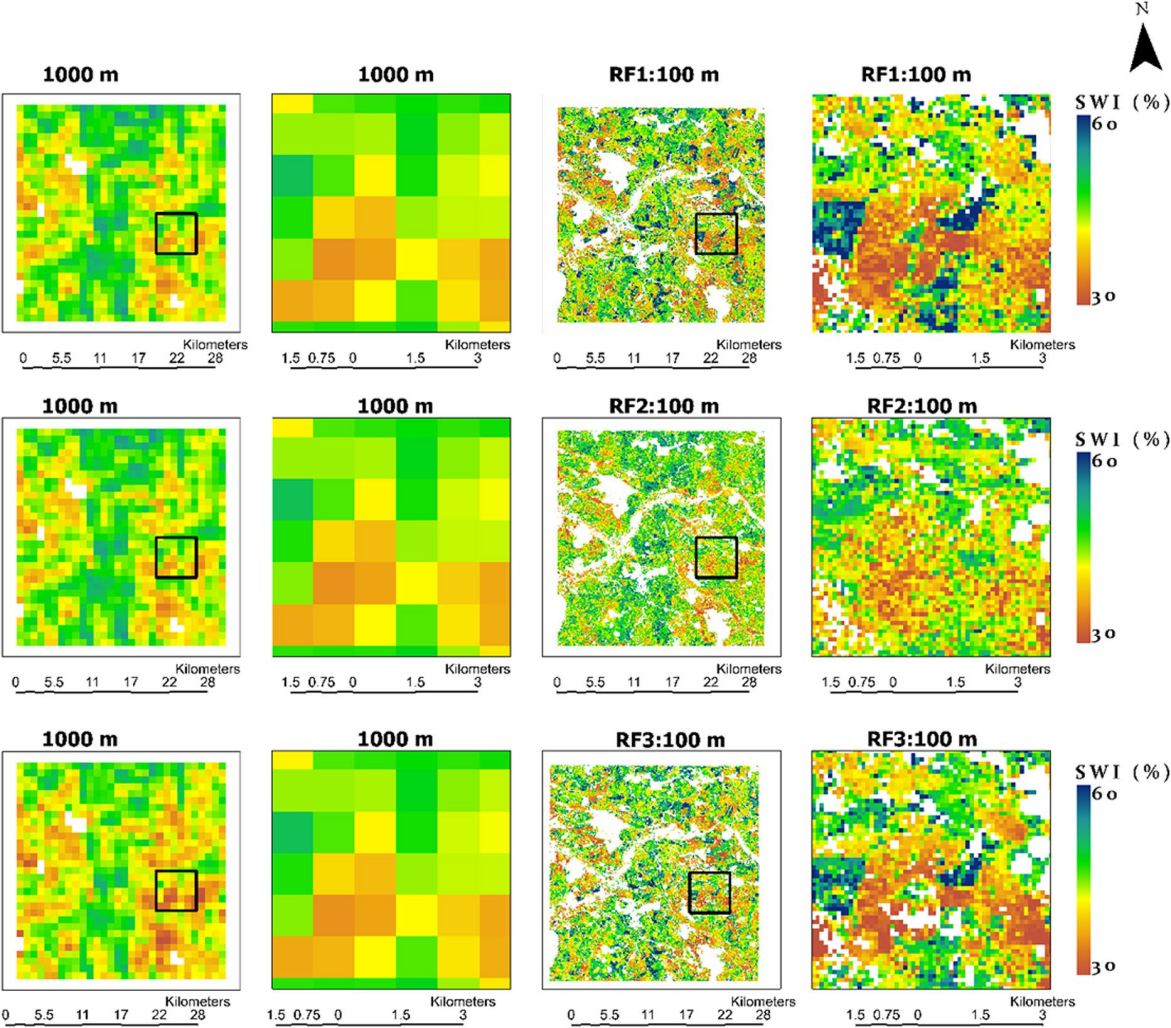
SWI_T-1km_ and downscaled SWI_T-100m_ with *T*_opt_ = 20 days using RF models used in this study. The black rectangles show the close in views of SWI_T-1km_ and SWI_T-100m_. RF, random forest; SWI_T-1km_, 1 km SWI dataset; SWI_T-100m_, 100 m downscaled SWI; *T*_opt_, optimized time length

Figure 12 (Appendix) displays the spatial distribution of SWI_T-1 km_ and SWI_T-100 m_ with *T*_opt_ (Table 2), on 26 June 2018. In addition, Fig. 7 presents the close-in views (black rectangle) to show the detailed spatial comparison between SWI_T-1 km_ and SWI_T-100 m_ with *T*_opt_ = 20 days. The date was chosen to ensure minimum cloud-affected Sentinel-2 acquisition for spatial comparison of all RF models. The spatial distribution of downscaled SWI_T-100 m_ is similar to the original SWI_T-1 km_, with more detailed information due to the increase in spatial resolution from 1 km to 100 m. The blank pixels indicate areas not covered by crops or grasslands.

**Fig. 8:**
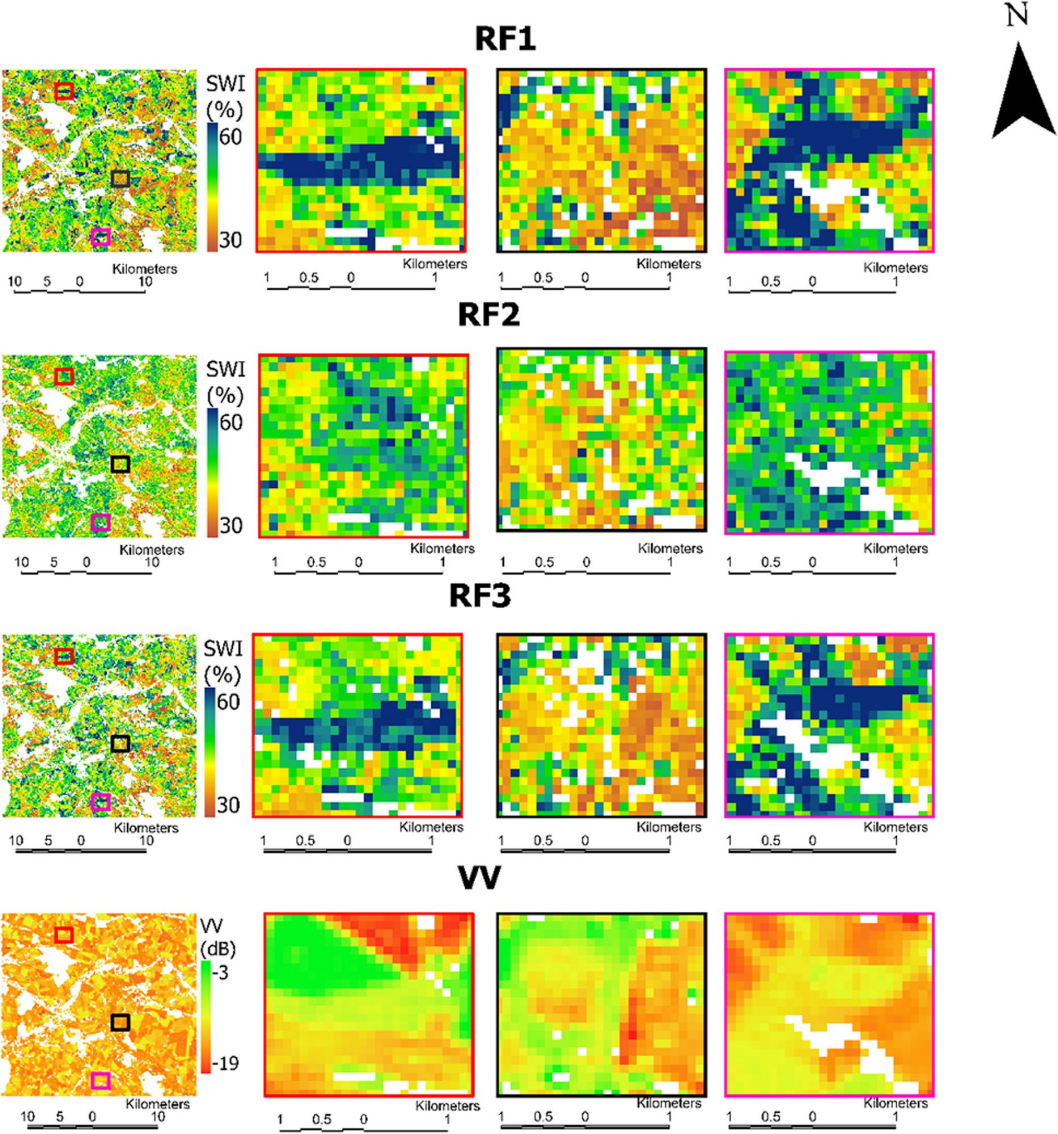
Zoom in views of SWI_T-100m_ with *T*_opt_ = 20, using RF models at three locations (red, black, and pink rectangles) along with *σ*_vv_^0^. RF, random forest; SWI_T-1km_, 1 km SWI dataset; SWI_T-100m_, 100 m downscaled SWI; *T*_opt_, optimized time length

Overall results show good spatial agreement between coarse and high-resolution downscaled results. However, the downscaled high-resolution results using RF1 and RF3 exhibit overestimation and underestimation at higher and lower values, respectively. Additionally, the RF1 and RF3 maps exhibit greater heterogeneity compared to the RF2 maps (Table 4). This is particularly evident at lower *T*_opt_ (i.e., 20 and 40 days), due to the greater importance of *σ*_vv_^0^ (Fig. 6) at these *T*_opt_ values. Figure 8 provides a closer look at SWI_T-100 m_ with *T*_opt_ = 20 days, at three specific locations spanning from north to south within the study area. It is worth noting that these locations are not detectable at 1 km resolution due to spatial aggregation. In addition, Fig. 8 also shows *σ*_vv_^0^ values at these locations. The pixels where SWI_T-100 m_ from RF1 and RF3 are overestimated correspond to higher *σ*_vv_^0^ values, while locations with underestimations correspond to lower *σ*_vv_^0^ values.

**Table 4:** Mean and standard deviation of SWIT_-1 km_ and RF downscaled SWI_T-100 m_ at agrometeorological locations. The *R* and RMSE between SWIT_-1 km_ and SWI_T-100 m_ are also provided

***T***_**opt**_**(days)**	**Mean (%)**		**SD (%)**		***R***	**RMSE (%)**	**Models**
	**1 km**	**100 m**	**1 km**	**100 m**			
20	50.2	51.3	17.8	19.4	0.94	6.63	RF1
	50.1	50.3	17.7	18.5	0.96	4.96	RF2
	50.2	50.9	17.8	20.3	0.95	5.64	RF3
40	51.9	52.9	17.5	18.5	0.94	5.91	RF1
	51.8	52.4	17.4	18.0	0.96	4.80	RF2
	51.9	52.6	17.5	19.6	0.95	5.23	RF3
60	53.8	54.5	16.5	17.2	0.94	5.17	RF1
	53.7	54.1	16.5	16.8	0.96	4.47	RF2
	53.8	54.2	16.5	18.2	0.95	4.78	RF3
100	56.9	57.2	13.8	14.1	0.94	4.89	RF1
	56.9	57.3	13.8	13.9	0.96	4.00	RF2
	56.9	57.3	13.8	15.1	0.95	4.18	RF3

### Spatial validation of downscaled SWI

**Fig. 9:**
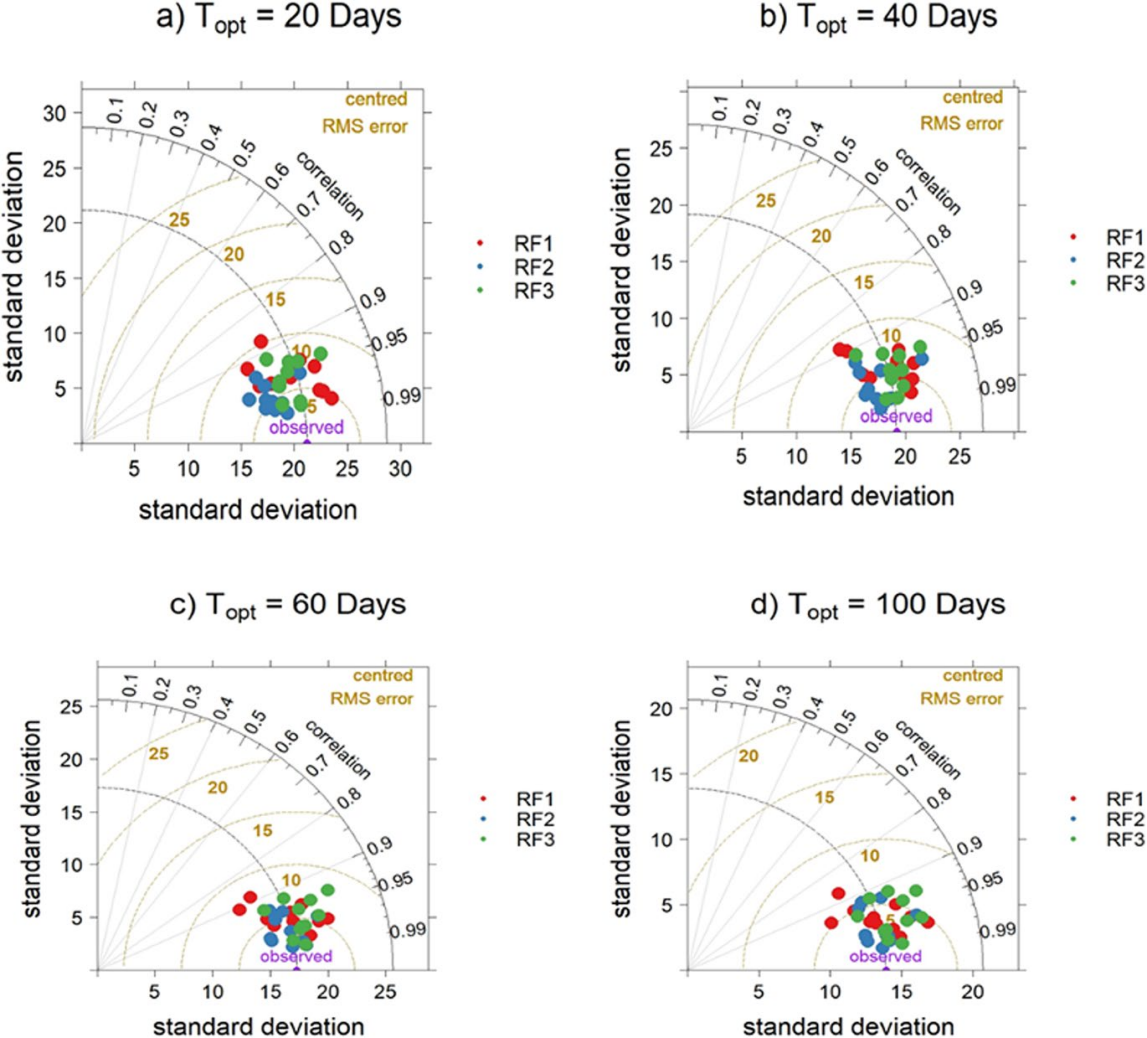
Spatial validation of different RF downscaled SWI_T-100m_ at agrometeorological stations. The black and brown dotted lines represent *R* and centered RMSE, respectively. RF, random forest; SWI_T-100m_, 100 m downscaled SWI; *T*_opt_: optimized time length

The spatial validation of the downscaled SWI_T-100 m_ was carried out by evaluating the pixel’s values of SWI_T-1 km_ and downscaled SWI_T-100 m_ at agrometeorological stations and presented in Fig. 9 as Taylor diagram (Taylor, 2001). This diagram combines *R*, centered RMSE, and standard deviation. The predictions that agree well lie nearest the purple observed point. The diagram shows that the *R* value between SWI_T-1 km_ and SWI_T-100 m_ was approximately over 0.90 at each agrometeorological station.

Table 4 presents a comparison of the mean and standard deviation (SD) of SWI_T-1 km_ and high-resolution SWI_T-100 m_ at agrometeorological stations (Fig. 1). The table also includes the *R* and RMSE between SWI_T-1 km_ and SWI_T-100 m_ at these locations. For comparing the RF models, we used only the acquisition dates (Supplementary Table S1) of SAR data used in RF3 to present the results of RF1 in Table 4. Nevertheless, the overall results align similarly. The slight difference between SWI_T-1 km_ values in RF2 and RF3 is due to the closer alignment of Sentinel-1 acquisition dates with the Sentinel-2 data used for training RF3. The mean values of SWI at *T*_opt_ = 20 days with 1 km (100 m) spatial resolution were 50.2 (51.3), 50.1 (50.3), and 50.2 (50.9) with RF1, RF2, and RF3, respectively. A similar trend is evident with other *T*_opt_ values. The difference between 1 km and 100 m mean values is lower with RF2 as compared to the RF1 and RF3. The small difference between original and downscaled results indicates a higher performance of the model. RF2 showed better performance on high-resolution prediction after the residual correction compared to RF1 and RF3. Similarly, RF3 provided an improvement over RF1. The SD values of SWI at 1 km (100 m) spatial resolution with *T*_opt_ = 20 days were 17.8 (19.4), 17.7 (18.5), and 17.8 (20.3) with RF1, RF2, and RF3, respectively. Across all three RF models and *T*_opt_ values, SWI_T-100 m_ maps at 100 m spatial resolution exhibited higher SDs compared to SWI_T-1 km_ maps. The difference between SD values SWI_T-100 m_ and SWI_T-1 km_ indicated greater spatial detail, variation, and heterogeneity with SWI_T-100 m_ as indicated in Fig. 12. RF3 showed higher spatial heterogeneity compared to RF1 and RF2 as presented in Table 4 with greater SD compared to RF1 and RF2.

The results in Table 4 also indicate high spatial accuracy (*R* ≥ 0.94) with all RF models. The spatial accuracy assessment reveals that RF2 slightly outperformed RF1 and RF3. The performance difference is higher at *T*_opt_ of 20 and 40 days. However, RF1 and RF3 exhibited more spatial heterogeneity and variation compared to RF2 as presented in Fig. 12. The results show that the use of optical and SAR data together is more of a spatial improvement on RF1. RF3 provided better spatial accuracy and comparison with SWI_T-1 km_ than RF1. Table 3 indicates a decrease in RMSE with an increase in *T*_opt_. The lowest RMSE values 4% and 4.17% were achieved with *T*_opt_ = 100 for RF2 and RF3, respectively, attributed to lower variability of RZSM with soil depth as previously mentioned. The spatial accuracy and distribution of RF1 are comparable to those of RF2 and RF3 after residual correction. This is important in the context of uninterrupted SWI_T-1 km_ downscaling due to the availability of Sentinel-1 data in all weather conditions.

*RF* random forest, *SWI*_*T-1 km*_ 1 km SWI dataset, *SWI*_*T-100 m*_ 100 m downscaled SWI, *T*_*opt*_ optimized time length.

### Estimation and validation of root zone soil moisture

**Fig. 10:**
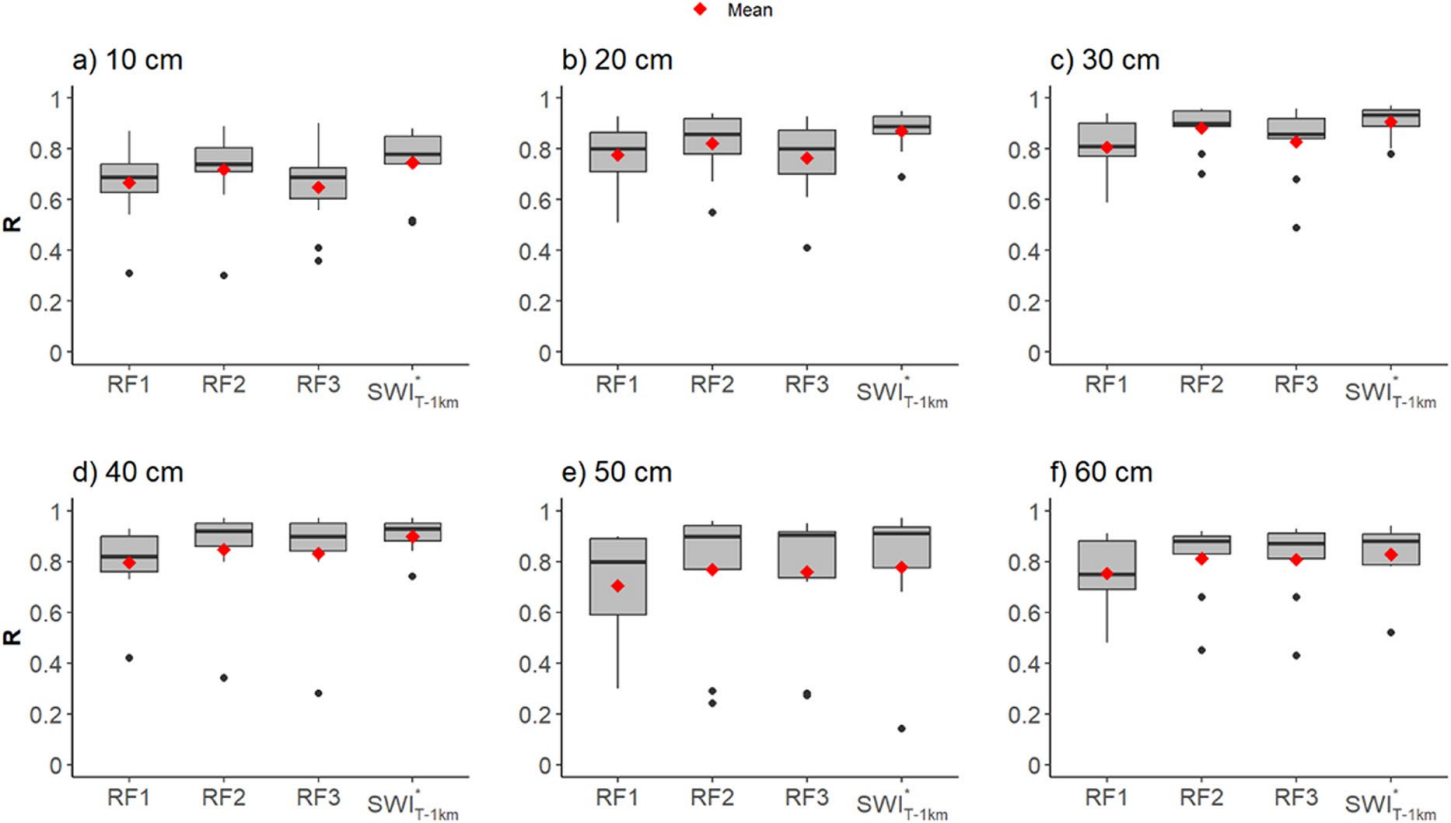
Box plots from station-wise *R* between in situ RZSM and SWI^*^_T_ (1 km and 100 m) with *T*_opt_. The black line in the middle indicates the median *R*, while black dots indicate outliers. RZSM, root zone soil moisture; SWI^*^_T-1km_, converted 1 km SWI dataset; SWI^*^_T-100m_, converted downscaled 100 m SWI; *T*_opt_, optimized time length

**Fig. 11:**
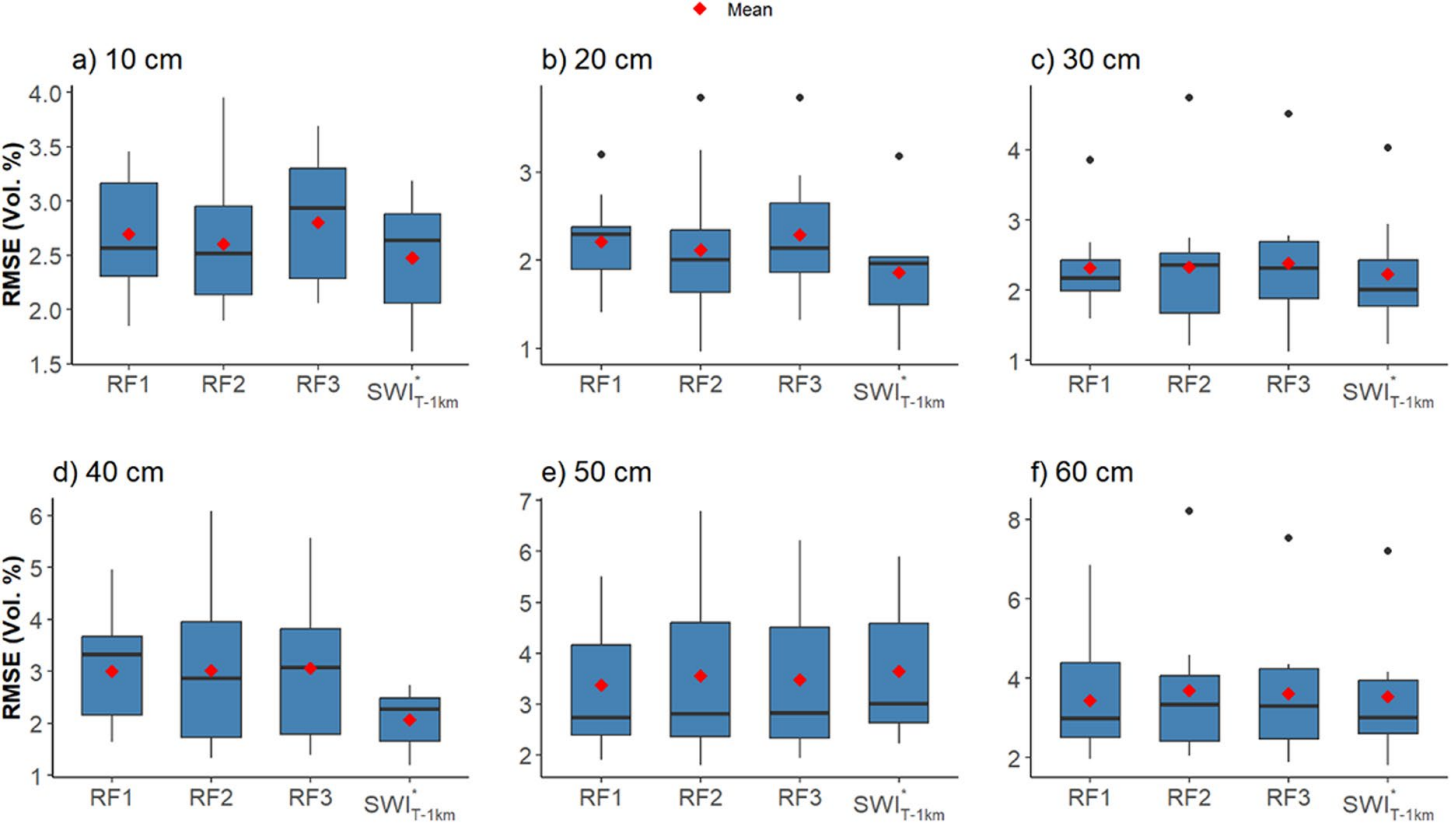
Box plots from station-wise RMSE between in situ RZSM and SWI^*^_T_ (1 km and 100 m) with *T*_opt_. The black line in the middle indicates the median RMSE, while black dots indicate outliers. RZSM, root zone soil moisture; SWI^*^_T-1km_, converted 1 km SWI dataset; SWI^*^_T-100m_, converted downscaled 100 m SWI; *T*_opt_, optimized time length

**Fig. 12:**
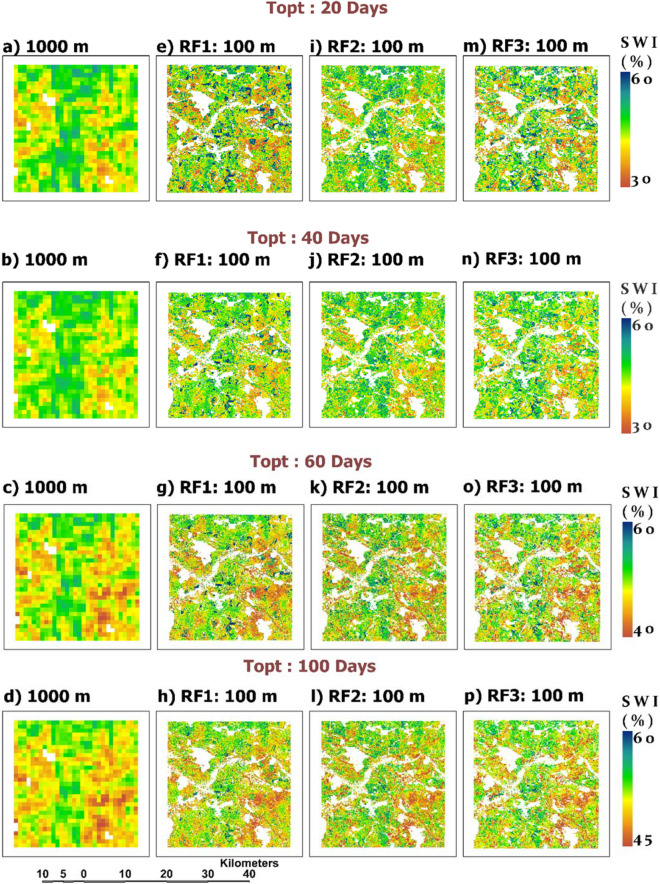
Spatial distribution of the SWI_T-1 km_ (**a**:**d**) and downscaled SWI_T-100 m_ with *T*_opt_; RF1(**e**:**h**), RF2 (**i**:**l**), and RF3 (**m**:**p**) on 26 June 2018. RF, random forest; SWI_T-1 km_, 1 km SWI dataset; *T*_opt_, optimized *T*

**Fig. 13:**
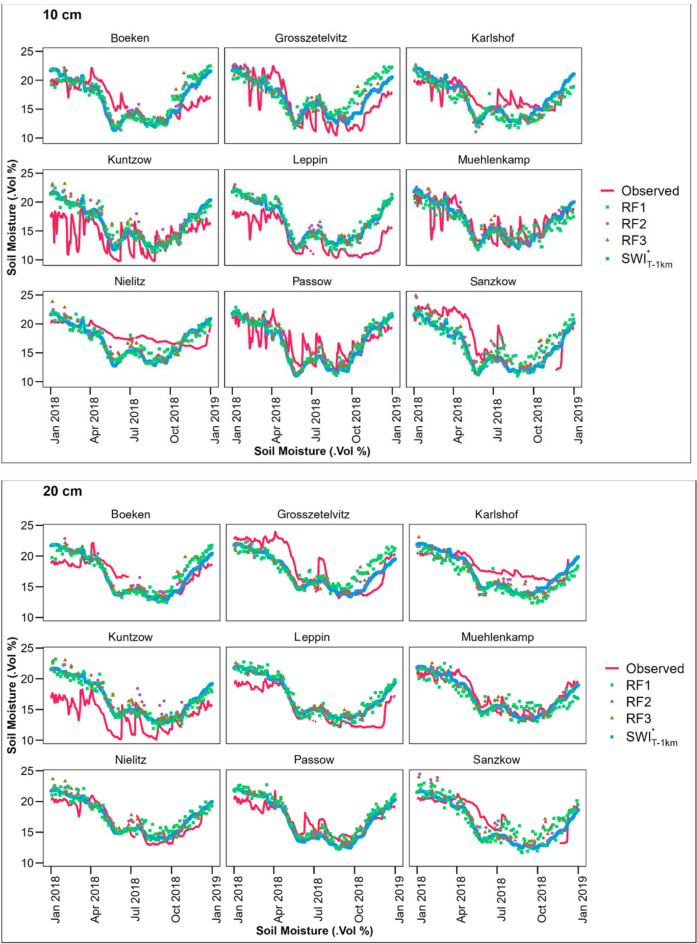

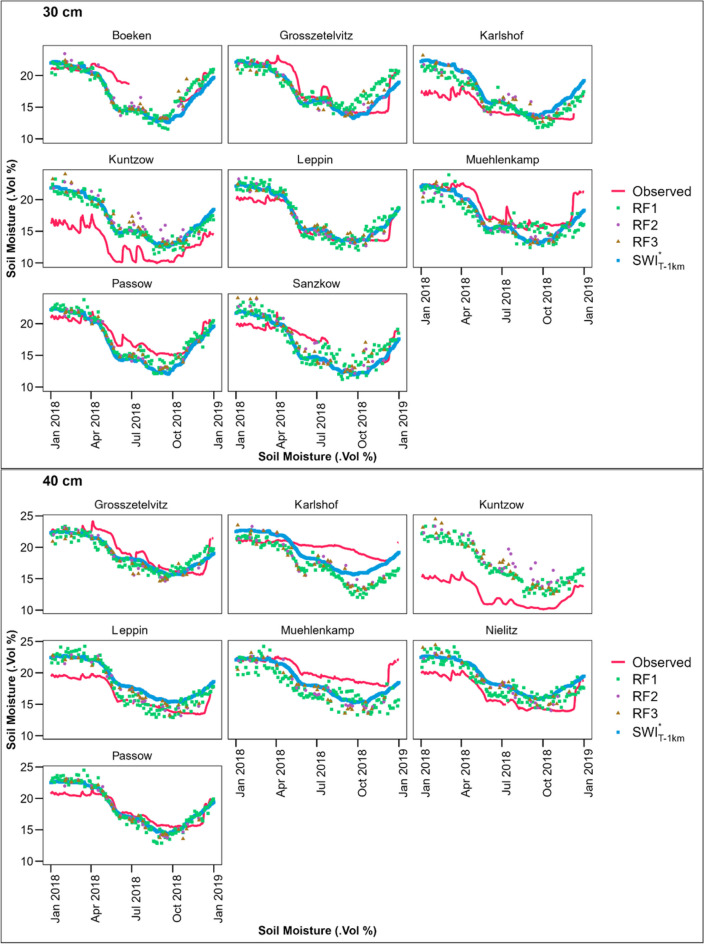

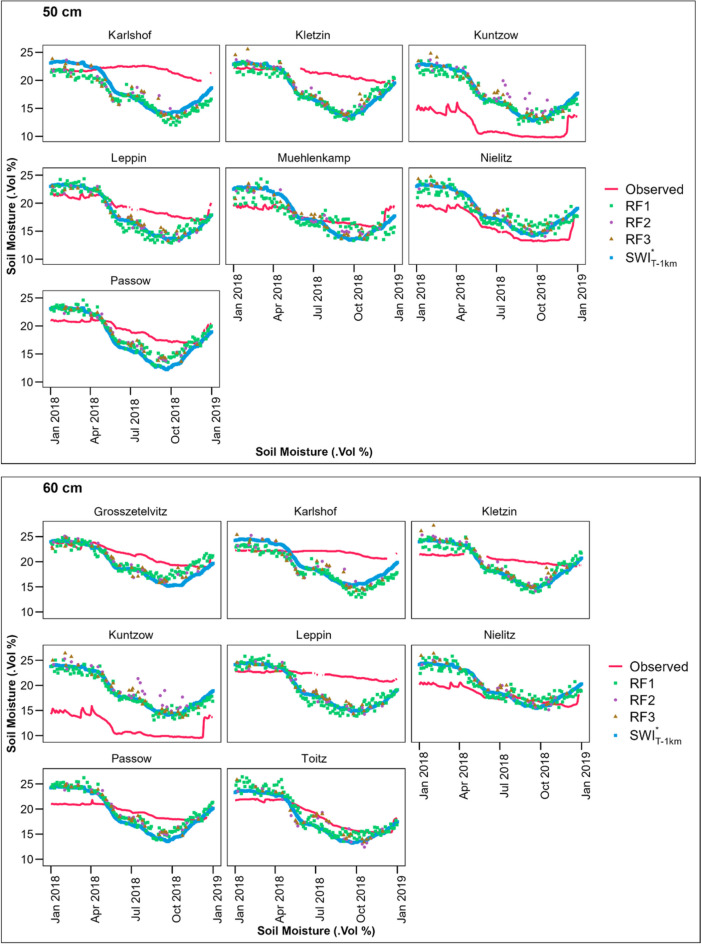
Temporal comparison between in situ SM and SWI.^*^ (1 km and 100 m) at each depth for available station in 2018

The SWI^*^ values (in Vol.%) were calculated using the *T*_opt_ datasets of SWI_T-1 km_ and RF downscaled SWI_T-100 m_, at 1 km and 100 m, respectively, referred as SWI^*^_T-1 km_ and SWI^*^_T-100 m_. Figures 10 and 11 display the depth-wise in situ validation (*R* and RMSE) at available agrometeorological stations during 2018, comparing observed RZSM and SWI^*^. The red dots represent the mean *R* and RMSE calculated by averaging the *R* and RMSE of individual stations. Figure 13 (Appendix) presents the depth-wise temporal comparison between observed RZSM and SWI^*^_T-1 km_ and SWI_T-100 m_ for individual stations with available data during 2018.

The value of *R* increases between 10 and 40 cm from a level of *R* = 0.65 to *R* > 0.8 (indicated by the means and medians). The median values are generally at the same level (*R* > 0.80) in deeper layers (> 30 cm); however, means are obviously decreased by outliers. The highest and lowest mean *R* values were achieved at 10 cm and 30 cm, respectively. The lower *R* compared to other depths is due to the inadequate capture of sudden changes in the observed RZSM at the 10 cm depth (Fig. 13 in the Appendix).

RF2 resulted in slightly better agreement with observed RZSM measurements as compared to RF1 and RF3, between 10 and 30 cm. RF2 achieved mean *R* 0.76 (10 cm) and 0.88 (30 cm) within agrometeorological stations as compared to RF3 (10 cm: 0.68; 30 cm: 0.82) and RF1 (10 cm: 0.67; 30 cm: 0.81). The mean *R* of RF3 and RF1 at soil depths of 10 to 30 cm across agrometeorological stations is comparable. However, at depths of 40 to 60 cm, RF3 exhibits slightly superior performance, achieving accuracy comparable to that of RF2 in terms of mean *R* results. SWI^*^_T-1 km_ outperforms SWI^*^_T-100 m_ at depths of 20 cm and 30 cm depths, while SWI^*^_T-100 m_ has slightly better accuracy in terms of mean *R* at 40 cm and 50 cm soil depths.

The RMSE between the observed RZSM and SWI^*^_T_ (1 km and 100 m) varies between 0.96 (Vol.%) (at 20 cm) and 8.2 (Vol.%) (at 60 cm), with its variability among agrometeorological stations increasing as the depth exceeds beyond 30 cm (Fig. 11). Consequently, from 10 to 30 cm, the mean and median *R* values demonstrate closer agreement, while beyond 30 cm, the mean RMSE is influenced by the variability of results among agrometeorological stations. RMSE (Vol.%) between observed RZSM and SWI^*^_T-1 km_ (SWI^*^_T-100 m_: RF1, RF2, RF3) ranges from 1.61 (1.85, 1.90, 2.06) to 3.47 (3.46, 3.96, 3.69) at 10 cm across agrometeorological stations, whereas at 60 cm, the RMSE (Vol.%) varies from 1.63 (1.97, 2.05, 1.88) to 7.1 (6.9, 8.20, 7.54). SWI^*^_T-1 km_ demonstrates slightly higher agreement compared to the downscaled SWI_T-100 m_, exhibiting a marginally lower RMSE. The RMSE difference between SWI^*^_T-1 km_ and SWI^*^_T-100 m_, where the RF model with the lowest RMSE is considered at each depth, ranges from 0.03 to 0.17 (Vol.%). This difference decreases as soil depth increases.

The temporal comparison (Supplementary Figure S1) between mean observed RZSM and SWI^*^_T_ (1 km and 100 m) at agrometeorological locations shows good agreement, especially within the 20 cm and 30 cm depths and during the summer months. However, during the winter period, both tend to overestimate the observed RZSM.

## Discussion

Local RZSM estimation and monitoring using satellite data is challenging due to the inability of this method to directly derive high-resolution RZSM. This work proposed a rapid scheme for the estimation of high-resolution RZSM at different depths using readily available SWI_T-1 km_. In the following, we discuss the control of *T*_opt_, and the depth specificity of SWI_T-1 km_ on RZSM estimation is explored below, highlighting their importance in the context of our proposed method and in comparison with previous studies. In addition, we discuss the effectiveness of optical and SAR variables for RF downscaling and examine their role in the improvement of RZSM accuracy and resolution. Afterwards, we compare the estimated RZSM and accuracy with the results of previous studies. Finally, we discuss the advantages of our approach, including its effectiveness and accessibility, as well as its limitations and areas for improvement in the future.

### Optimum time length and depth specificity

The calibration and validation of the *T* parameter associated with the SWI_T-1 km_ dataset to select *T*_opt_ is the first step to attain good performance in estimating RZSM. The calibration of eight *T* values associated with SWI_T-km_ dataset against in situ RZSM demonstrated decent performance with *R* ranges between − 0.38 and 0.97 with an average value of 0.59 (Fig. 4). However, these results for DEMMIN are consistent with previous studies (C Albergel et al., 2012; Grillakis et al., 2021; Paulik et al., 2014) conducted in different regions and with different datasets, ranging in depth from localized point measurements to broader satellite-derived data. Also, the observed increased trend in *T*_opt_ values with an increase in soil depth (Fig. 5 and Table 2) agrees with previous studies (Albergel et al., 2009; Brocca et al., 2009, 2010; Ceballos et al., 2005; Paulik et al., 2014) that were conducted at different spatial resolutions in different soil climatic regions and demonstrated variability in *T*_opt_ within the same soil depth range.

The consistent accuracy levels underpin that the EF approach with the single parameter *T* is easy to calibrate. However, *T*_opt_ variations challenge physical explanations (Albergel et al., 2008; Ceballos et al., 2005). For instance, Brocca et al. (2010) selected *T*_opt_ values of 19.5, 23, and 29 days for soil depths of 10, 20, and 40 cm, respectively, in the Mediterranean climate with a mean annual rainfall of 950 mm. We also selected *T*_opt_ of 20 days for 10 cm, while *T*_opt_ values for 20 cm and 40 cm exceeded those elaborated by Brocca et al. (2010). This may be due to comparatively lower mean annual rainfall at Demmin (500–600 mm), which is associated with higher *T*_opt_ (Albergel et al., 2008; Wang et al., 2017; Yang et al., 2022), and the use of discrete fixed intervals of *T* with CGLS SWI_T-1 km_ dataset. Another possible reason is the presence of vegetation which generally increases *T*_opt_ (T. Wang et al., 2017). From the model perspective, higher *T*_opt_ values indicate that RZSM at time *t*_n_ relies less on SSM at *t*_n_ but more on previous RZSM at *t*_n-1_(Yang et al., 2022). The selected *T*_opt_ values in this study are closer to those found by Ceballos et al. (2005) in the Duero basin (Spain). They reported *T*_opt_ values of 40 days and 60 days for soil layers 0–25 cm and 50–100 cm, respectively. The Duero basin shares similar characteristics with DEMMIN, including mean annual rainfall ranging between 300 and 600 mm and sandy to sandy loam soil texture (Martínez-Fernández et al., 2021).

Grillakis et al. (2021) compared the SWI calculated from the ESA Climate Change Initiative (CCI) with the in situ measurements from 353 International Soil Moisture Network (ISMN) locations (Dorigo et al., 2011). They obtained *T*_opt_ values ranging from 7 to 46 for median and an average depth of 35 cm and 22 cm, respectively. In addition, the presented study demonstrated the applicability of the calibration approach used in this study by the overall performance of the SWI_T-1 km_ against in situ RZSM.

### Effect of optical and SAR variables for RF downscaling

The feature importance results indicate that NDWI is more important for the performance of the RF models in downscaling SWI than other SAR and optical variables (Fig. 6). This is consistent with the findings by Hegazi et al. (2023), who reported that NDWI is more sensitive than NDVI and GVMI, and in combination, these indices outperform the single Sentinel-2 bands. This is because indices are calculated by combining two or more bands (Hegazi et al., 2023). RF modeling at lower depths with *T*_opt_ of 20 and 40 days indicates that *σ*_vv_^0^ was more important than NDVI. This is because backscatter coefficients are more sensitive to SSM and roughness, which have a greater impact at shallower depths. As depth increases, the influence of surface conditions diminishes, reducing the relevance of the backscatter coefficient. This observation confirms previous studies (Baghdadi et al., 2017; Hajj et al., 2017) that have also reported the ability of SAR data, particularly *σ*_vv_^0^ in estimating surface water content. Moreover, the importance of *σ*_vv_^0^ was also found to be more influential than *σ*_vH_^0^. For instance, Baghdadi et al. (2017) found that *σ*_vv_^0^ is more sensitive to soil moisture *t* and less effected by vegetation and surface roughness as compared to *σ*_vH_^0^. In response to the decrease in importance of *σ*_vv_^0^, the importance of GVMI slightly increased. However, the importance of NDVI was the same level of variable importance but was more than *σ*_vv_^0^, with an increase in *T*_opt_ to 100 days.

The spatial accuracy of the high-resolution SWI_T-100 m_ obtained with RF1 and RF3 was slightly lower than achieved with RF2 (Table 4), most likely due to the influence of surface roughness on the backscatter coefficient (Fig. 8). The use of the backscatter coefficient in RF1 and RF3 resulted in higher SD than RF2. The higher SD in high-resolution prediction indicates greater spatial variation and heterogeneity (Fathololoumi et al., 2022). The effect of surface roughness for soil moisture downscaling using high-resolution SAR backscatter data is also reported in other studies (Bryant et al., 2007; Peng et al., 2017). This effect was not present at 1 km resolution, possibly due to narrow value ranges related to smoothing effects of the applied spatial aggregation. This effect is more pronounced in RF1 than RF3 (Fig. 8), because the inclusion of optical vegetation indices improved the spatial accuracy of RF3. The latter underlines the value of the NDVI for reducing uncertainty introduced by surface roughness when only SAR data are utilized for downscaling as previously also indicated by Hajj et al. (2017).Vegetation indices further express different vegetation conditions and are recommended to downscale soil moisture (Bai et al., 2019) and to further enhance the accuracy of downscaling process when synergistically using SAR and optical data.

### Root zone soil moisture estimation and comparison

The comparison between SWI^*^_T_ at 1 km and 100 m resolution and the in situ depth-wise RZSM shows that SWI^*^_T-1 km_ exhibits slightly better agreement with observed RZSM from 10 to 40 cm. This can simply be attributed to the tendency of downscaled results to retain the characteristics of the predictors used, leading to some missed temporal changes (Qu et al., 2021). Merlin et al. (2013) also reported that high-resolution soil moisture may not always provide better accuracy than coarse resolution due to landscape heterogeneity. The results in this study illustrated that SWI^*^_T_ both spatial resolutions are in better agreement and representative with observed RZSM values at 20 cm and 30 cm. In contrast, deeper observations show higher variability of *R* and RMSE among agrometeorological stations. Brocca et al. (2010) similarly reported a decline in *R* from 0.67 (10 cm) to 0.61 (40 cm) between RZSM and the 25 km SW^*^_T_ dataset obtained from ASCAT backscatter observations. Furthermore, the average RMSE for RF-based downscaled SWI^*^_T-100 m_ at 20 cm ranges from 2 (Vol.%) to 2.23 (Vol.%). These results resemble those obtained by Ceballos et al. (2005), who found an RMSE of 2.4 (Vol.%) for the 0–25 cm layer. Moreover, the average *R* obtained with SWI^*^_T-100 m_ among agrometeorological sites at 20 cm depth was ~ 0.80, in turn agreeing with the findings of Brocca et al. (2009), who reported a comparable mean *R* of 0.81 for SW^*^_T_ in representing RZSM at a depth of 15 cm.

The mean SWI^*^_T_ (1 km and 100 m) and observed RZSM across agrometeorological locations showed higher agreement during the summer season. Both datasets exhibited an overestimation of the in situ data in winter. Similarly, Fathololoumi et al. (2022) received increased RMSE during the cold season between 30 m resolution CGLS SWI and SSM in their analysis in the USA, France, and Iran.

### Advantages and limitations

The CGLS SWI_T-1 km_ offers a solution in regions with reduced availability of in situ RZSM observations. The long-term spatial information on soil moisture can aid in identifying areas experiencing agricultural drought due to soil moisture shortage (Piedallu et al., 2013). To monitor local RZSM variations, downscaled SWI_T-100 m_ data can provide more spatial details and localized information. Under smart agricultural initiatives, such estimation schemes can be effectively utilized to monitor agricultural water demand, e.g., for irrigation monitoring or scheduling.

The outstanding temporal and spatial resolution of SAR data from Sentinel satellites provides consistency in the availability of high-resolution SWI datasets. However, while the combined use of optical and SAR data offers superior results, it may not always be readily available. The temporal and spatial reconstruction of missing information in optical data offers an opportunity to combine optical and SAR data at a higher temporal resolution, utilizing the capabilities of both sensors to obtain high-resolution and accurate results (Q. Wang et al., 2023). Additionally, this approach could allow the incorporation of other high-resolution optical and thermal satellite data such as Landsat, which has a lower temporal resolution (~ 16 days) compared to Sentinel-2.

The CGLS SWI_T-1 km_ with eight fixed *T* values may not always accurately represent the dynamic nature of soil moisture at lesser depths. Although only one parameter *T* is needed to calibrate EF, saving computational time, the physical explanation of *T* needs further consideration. The parameter *T* has been found to be related to the factors that influence soil moisture dynamics, such as evapotranspiration, hydraulic properties, soil thickness, and strata (Ceballos et al., 2005). In addition, the use of the high resolution of environmental, topographic, and soil property variables for pixel-wise *T* calibration can further optimize the variability caused by topography, soil, and hydraulic properties in the region (Yang et al., 2022). Taking these factors into account will further improve the reliability of the calibration process for the CGLS SWI_T-1 km_ dataset. Furthermore, we used constant *T*_opt_ values across the entire study area to estimate RZSM at specific soil depths ranging from 10 to 60 cm. However, using constant *T*_opt_ may lead to over-smoothing in the estimated values, as observed in our estimates at 10 cm depth as well (Fig. 13 in the Appendix). The use of variable *T*_opt_ can improve accuracy in the estimation of RZSM observations as reported by Herbert et al. (2020).

We utilized the 1 km CGLS SWI, derived from the fusion of 25 km ASCAT and 1 km Sentinel-1 SSM (Bauer-Marschallinger et al., 2020), which is available for Europe, while the global product, based solely on ASCAT SSM, is available at 12.5 km resolution. However, 1 km data is still not sufficient for localized agricultural applications such as irrigation management and water stress yield. Further downscaling of the improved 1 km resolution data may preserve boxy artifacts introduced during the initial spatial resolution improvement, a common issue highlighted by Merlin et al. (2013). Nonetheless, Ojha et al. (2019) have reported that high spatial resolution predictions achieved through sequential downscaling can capture the heterogeneity in soil moisture estimates. The decision to use 1 km improved CGLS SWI data was made because the calibration of *T*_opt_ values requires high-quality and representative in situ data, which in this study was limited to DEMMIN. Using the 10 km resolution data would restrict the availability of SWI data for the calibration of *T*_opt_. Additionally, the accuracy achieved in this study demonstrates the robustness of the methods used. Furthermore, the method employed in this study is simple and can be applied to other areas using the 10 km global SWI product to estimate high-resolution RZSM at different soil depths.

In situ observations, such as those found in regions like DEMMIN, are crucial for the further transfer of the method. However, in regions where in situ data is unavailable, reanalysis products such as ERA5 and Global Land Data Assimilation System (GLDAS) could be considered. Despite their potential, these datasets have inherent limitations. The coarser spatial resolution may hinder the accurate capture of localized soil moisture variations, especially in areas with heterogeneous landscapes. Secondly, it is crucial to carefully select datasets based on their performance in specific regions, as there may be performance differences (Zheng et al., 2022). Therefore, although these datasets provide a valuable workaround, it is still crucial to address these limitations to ensure the accuracy and reliability of soil moisture estimation. This is particularly important in applications crucial for agricultural management, such as irrigation monitoring and scheduling.

## Conclusions

The presented study demonstrates the utilization of various input dataset combinations for RF-based downscaling of the 1 km CGLS SWI dataset and the estimation of high-resolution RZSM at different depths at the example of the intensively used agricultural landscape in Mecklenburg-Western Pomerania, Germany (DEMMIN).

The eight different *T* values provided with the CGLS SWI_T-1 km_ dataset offered the opportunity for the selection of *T*_opt_ that represents the RZSM at specific depths. CGLS SWI_T-1 km_ data showed reasonable agreement with the observed RZSM across all depths (*R* > 0.5 for 70% of the time series at agrometeorological stations). As expected, increases of *T*_opt_ with root zone depth indicate the downward directed processes in soil moisture dynamics in the root zone of the observed agricultural landscape.

To generate high-resolution RZSM from CGLS SWI_T-1 km_ data, RF was trained using multisource geodata from optical (Sentinel-2), SAR (Sentinel-1), and topographic (SRTM) variables. The results showed that the RF downscaling method has strong applicability in the area and downscaled results after residual correction include more spatial details and can better represent the spatial distribution of RZSM. Variable importance analysis, combined with performance assessments, highlighted the significant role of remote sensing features. NDWI was consistently identified as the most critical feature across all soil depths. At shallower depths, the backscatter coefficient in VV polarization (*σ*_vv_^0^) demonstrated considerable importance. Conversely, as soil depth increased, the significance of optical variables became more pronounced, indicating their growing influence on RF modeling with increasing soil depth. Overall, it can be concluded that incorporating both optical and SAR data leads to better predictions on test sets and outperforms their individual use in RF training. Validation of the RZSM was performed against a wide range of ground observations at 11 agrometeorological sites and showed good accuracy, notably at 20 cm and 30 cm depths, exhibiting consistent correlation across agrometeorological stations with lower RMSE values. The downscaled SWI_T-100 m_ provides higher spatial detail with negligible accuracy differences. These findings collectively emphasize the utility and accuracy of CGLS SWI_T-100 m_ datasets for RZSM monitoring and underscore the potential of high-resolution data for improving agricultural and hydrological management practices.

## Supplementary Information

Supplementary Material 1

## Data Availability

The satellite and ground data are obtained through open-source platforms Google Earth Engine, Copernicus Data Services, and TERENO. Further inquiries can be directed to the corresponding author.

## References

[CR1] Albergel, C., Rüdiger, C., Pellarin, T., Calvet, J. C., Fritz, N., Froissard, F., et al. (2008). From near-surface to root-zone soil moisture using an exponential filter: An assessment of the method based on in-situ observations and model simulations. *Hydrology and Earth System Sciences,**12*(6), 1323–1337. 10.5194/hess-12-1323-2008 10.5194/hess-12-1323-2008

[CR2] Albergel, C., Rüdiger, C., Carrer, D., Calvet, J. C., Fritz, N., Naeimi, V., et al. (2009). An evaluation of ASCAT surface soil moisture products with in-situ observations in Southwestern France. *Hydrology and Earth System Sciences,**13*(2), 115–124. 10.5194/hess-13-115-2009 10.5194/hess-13-115-2009

[CR3] Albergel, C., de Rosnay, P., Gruhier, C., Muñoz-Sabater, J., Hasenauer, S., Isaksen, L., et al. (2012). Evaluation of remotely sensed and modelled soil moisture products using global ground-based in situ observations. *Remote Sensing of Environment,**118*, 215–226. 10.1016/j.rse.2011.11.017 10.1016/j.rse.2011.11.017

[CR4] Albergel, C., Dorigo, W., Reichle, R. H., Balsamo, G., Derosnay, P., Muñoz-sabater, J., et al. (2013). Skill and global trend analysis of soil moisture from reanalyses and microwave remote sensing. *Journal of Hydrometeorology,**14*(4), 1259–1277. 10.1175/JHM-D-12-0161.1 10.1175/JHM-D-12-0161.1

[CR5] Albergel, C., Zheng, Y., Bonan, B., Dutra, E., Rodríguez-Fernández, N., Munier, S., et al. (2020). Data assimilation for continuous global assessment of severe conditions over terrestrial surfaces. *Hydrology and Earth System Sciences,**24*(9), 4291–4316. 10.5194/hess-24-4291-2020 10.5194/hess-24-4291-2020

[CR6] Babaeian, E., Sadeghi, M., Jones, S. B., Montzka, C., Vereecken, H., & Tuller, M. (2019). Ground, proximal, and satellite remote sensing of soil moisture. *Reviews of Geophysics,**57*(2), 530–616. 10.1029/2018RG000618 10.1029/2018RG000618

[CR7] Babaeian, E., Paheding, S., Siddique, N., Devabhaktuni, V. K., & Tuller, M. (2021). Estimation of root zone soil moisture from ground and remotely sensed soil information with multisensor data fusion and automated machine learning. *Remote Sensing of Environment,**260*(March), 112434. 10.1016/j.rse.2021.112434 10.1016/j.rse.2021.112434

[CR8] Baghdadi, N., Hajj, M. E., Zribi, M., & Bousbih, S. (2017). Calibration of the water cloud model at C-band for winter crop fields and grasslands. *Remote Sensing,**9*(9), 1–13. 10.3390/rs9090969 10.3390/rs9090969

[CR9] Bai, J., Cui, Q., Zhang, W., & Meng, L. (2019). An approach for downscaling SMAP soil moisture by combining Sentinel-1 SAR and MODIS data. *Remote Sensing,**11*(23), 1–20. 10.3390/rs11232736 10.3390/rs11232736

[CR10] Bauer-Marschallinger, B., Paulik, C., Hochstöger, S., Mistelbauer, T., Modanesi, S., Ciabatta, L., et al. (2018). Soil moisture from fusion of scatterometer and SAR: Closing the scale gap with temporal filtering. *Remote Sensing,**10*(7), 1–26. 10.3390/rs10071030 10.3390/rs10071030

[CR11] Bauer-Marschallinger, B., Lacaze, R., & Cherlet, M. (2020). Copernicus global land operations “Vegetation and Energy”. CGLOPS-1, Framework Service Contract 199494 (JRC); Validation Report. Soil Water Index Collection 1km. Version 1.0. https://land.copernicus.eu/en/technical-library/validation-report-soil-water-index-version-1

[CR12] Breiman, L., Friedman, J, Stone, C. J. & Olshen, R. A. (1984). *Classification and regression trees*. (1st ed.). Taylor & Francis. 10.1201/9781315139470

[CR13] Brocca, L., Melone, F., Moramarco, T., & Morbidelli, R. (2009). Antecedent wetness conditions based on ERS scatterometer data. *Journal of Hydrology,**364*(1–2), 73–87. 10.1016/j.jhydrol.2008.10.007 10.1016/j.jhydrol.2008.10.007

[CR14] Brocca, L., Melone, F., Moramarco, T., Wagner, W., & Hasenauer, S. (2010). ASCAT soil wetness index validation through in situ and modeled soil moisture data in Central Italy. *Remote Sensing of Environment,**114*(11), 2745–2755. 10.1016/j.rse.2010.06.009 10.1016/j.rse.2010.06.009

[CR15] Brocca, L., Hasenauer, S., Lacava, T., Melone, F., Moramarco, T., Wagner, W., et al. (2011). Soil moisture estimation through ASCAT and AMSR-E sensors: An intercomparison and validation study across Europe. *Remote Sensing of Environment,**115*(12), 3390–3408. 10.1016/J.RSE.2011.08.003 10.1016/J.RSE.2011.08.003

[CR16] Bryant, R., Moran, M. S., Thoma, D. P., Holifield Collins, C. D., Skirvin, S., Rahman, M., et al. (2007). Measuring surface roughness height to parameterize radar backscatter models for retrieval of surface soil moisture. *IEEE Geoscience and Remote Sensing Letters,**4*(1), 137–141. 10.1109/LGRS.2006.887146 10.1109/LGRS.2006.887146

[CR17] Carlson, T. N., & Ripley, D. A. (1997). On the relation between NDVI, fractional vegetation cover, and leaf area index. *Remote Sensing of Environment,**62*(3), 241–252. 10.1016/S0034-4257(97)00104-1 10.1016/S0034-4257(97)00104-1

[CR18] Ceballos, A., Scipal, K., Wagner, W., & Martínez-Fernández, J. (2005). Validation of ERS scatterometer-derived soil moisture data in the central part of the Duero Basin, Spain. *Hydrological Processes,**19*(8), 1549–1566. 10.1002/hyp.5585 10.1002/hyp.5585

[CR19] Ceccato, P., Flasse, S., & Grégoire, J. M. (2002). Designing a spectral index to estimate vegetation water content from remote sensing data: Part 1: Theoretical approach. *Remote Sensing of Environment,**82*(2–3), 188–197. 10.1016/S0034-4257(02)00037-8 10.1016/S0034-4257(02)00037-8

[CR20] Chauhan, N. S., Miller, S., & Ardanuy, P. (2003). Spaceborne soil moisture estimation at high resolution: A microwave-optical/IR synergistic approach. *International Journal of Remote Sensing,**24*(22), 4599–4622. 10.1080/0143116031000156837 10.1080/0143116031000156837

[CR21] Chen, S., She, D., Zhang, L., Guo, M., & Liu, X. (2019). Spatial downscaling methods of soil moisture based on multisource remote sensing data and its application. *Water (switzerland),**11*(7), 1–25. 10.3390/w11071401 10.3390/w11071401

[CR22] Clark, M. P., Rupp, D. E., Woods, R. A., Zheng, X., Ibbitt, R. P., Slater, A. G., et al. (2008). Hydrological data assimilation with the ensemble Kalman filter: Use of streamflow observations to update states in a distributed hydrological model. *Advances in Water Resources,**31*(10), 1309–1324. 10.1016/j.advwatres.2008.06.005 10.1016/j.advwatres.2008.06.005

[CR23] Conrad, O., Bechtel, B., Bock, M., Dietrich, H., Fischer, E., Gerlitz, L., et al. (2015). System for automated geoscientific analyses (SAGA) v. 2.1.4. *Geoscientific Model Development,**8*(7), 1991–2007. 10.5194/gmd-8-1991-2015 10.5194/gmd-8-1991-2015

[CR24] Dorigo, W. A., Wagner, W., Hohensinn, R., Hahn, S., Paulik, C., Xaver, A., et al. (2011). The International Soil Moisture Network: A data hosting facility for global in situ soil moisture measurements. *Hydrology and Earth System Sciences,**15*(5), 1675–1698. 10.5194/hess-15-1675-2011 10.5194/hess-15-1675-2011

[CR25] El Hajj, M., Baghdadi, N., & Zribi, M. (2019). Comparative analysis of the accuracy of surface soil moisture estimation from the C- and L-bands. *International Journal of Applied Earth Observation and Geoinformation,**82*(June), 101888. 10.1016/j.jag.2019.05.021 10.1016/j.jag.2019.05.021

[CR26] Ermida, S. L., Soares, P., Mantas, V., Göttsche, F. M., & Trigo, I. F. (2020). Google earth engine open-source code for land surface temperature estimation from the Landsat series. *Remote Sensing,**12*(9), 1–21. 10.3390/RS12091471 10.3390/RS12091471

[CR27] Fathololoumi, S., Vaezi, A. R., Alavipanah, S. K., Ghorbani, A., & Biswas, A. (2020). Comparison of spectral and spatial-based approaches for mapping the local variation of soil moisture in a semi-arid mountainous area. *Science of the Total Environment,**724*, 138319. 10.1016/j.scitotenv.2020.138319 32408464 10.1016/j.scitotenv.2020.138319

[CR28] Fathololoumi, S., Karimi Firozjaei, M., & Biswas, A. (2022). Improving spatial resolution of satellite soil water index (SWI) maps under clear-sky conditions using a machine learning approach. *Journal of Hydrology,**615*(PA), 128709. 10.1016/j.jhydrol.2022.128709 10.1016/j.jhydrol.2022.128709

[CR29] Firozjaei, M. K., Fathololoumi, S., Alavipanah, S. K., Kiavarz, M., Vaezi, A. R., & Biswas, A. (2020). A new approach for modeling near surface temperature lapse rate based on normalized land surface temperature data. *Remote Sensing of Environment,**242*, 111746. 10.1016/j.rse.2020.111746 10.1016/j.rse.2020.111746

[CR30] Ford, T. W., Harris, E., & Quiring, S. M. (2014). Estimating root zone soil moisture using near-surface observations from SMOS. *Hydrology and Earth System Sciences,**18*(1), 139–154. 10.5194/hess-18-139-2014 10.5194/hess-18-139-2014

[CR31] Gao, B. C. (1996). NDWI—A normalized difference water index for remote sensing of vegetation liquid water from space. *Remote Sensing of Environment,**58*(3), 257–266. 10.1016/S0034-4257(96)00067-3 10.1016/S0034-4257(96)00067-3

[CR32] Grillakis, M. G., Koutroulis, A. G., Alexakis, D. D., Polykretis, C., & Daliakopoulos, I. N. (2021). Regionalizing root-zone soil moisture estimates from ESA CCI soil water index using machine learning and information on soil, vegetation, and climate. *Water Resources Research,**57*(5), 1–22. 10.1029/2020WR029249 10.1029/2020WR029249

[CR33] Guo, X., Fang, X., Zhu, Q., Jiang, S., Tian, J., Tian, Q., & Jin, J. (2023). Estimation of root-zone soil moisture in semi-arid areas based on remotely sensed data. *Remote Sensing,**15*(8), 2003. 10.3390/RS15082003 10.3390/RS15082003

[CR34] Hajj, M. E., Baghdadi, N., Zribi, M., & Bazzi, H. (2017). Synergic use of Sentinel-1 and Sentinel-2 images for operational soil moisture mapping at high spatial resolution over agricultural areas. *Remote Sensing,**9*(12), 1–28. 10.3390/rs9121292 10.3390/rs9121292

[CR35] He, L., Hong, Y., Wu, X., Ye, N., Walker, J. P., & Chen, X. (2018). Investigation of SMAP active-passive downscaling algorithms using combined Sentinel-1 SAR and SMAP radiometer data. *IEEE Transactions on Geoscience and Remote Sensing,**56*(8), 4906–4918. 10.1109/TGRS.2018.2842153 10.1109/TGRS.2018.2842153

[CR36] Hegazi, E. H., Samak, A. A., Yang, L., Huang, R., & Huang, J. (2023). Prediction of soil moisture content from Sentinel-2 images using convolutional neural network (CNN). *Agronomy,**13*(3), 1–18. 10.3390/agronomy13030656 10.3390/agronomy13030656

[CR37] Herbert, C., Pablos, M., Vall-llossera, M., Camps, A., & Martínez-Fernández, J. (2020). Analyzing spatio-temporal factors to estimate the response time between SMOS and in-situ soil moisture at different depths. *Remote Sensing,**12*(16), 2614. 10.3390/RS12162614 10.3390/RS12162614

[CR38] Heupel, K., Spengler, D., & Itzerott, S. (2018). A progressive crop-type classification using multitemporal remote sensing data and phenological information. *PFG - Journal of Photogrammetry, Remote Sensing and Geoinformation Science,**86*(2), 53–69. 10.1007/s41064-018-0050-7 10.1007/s41064-018-0050-7

[CR39] Holtgrave, A. K., Förster, M., Greifeneder, F., Notarnicola, C., & Kleinschmit, B. (2018). Estimation of soil moisture in vegetation-covered floodplains with Sentinel-1 SAR data using support vector regression. *PFG - Journal of Photogrammetry, Remote Sensing and Geoinformation Science,**86*(2), 85–101. 10.1007/s41064-018-0045-4 10.1007/s41064-018-0045-4

[CR40] Hosseini, M., McNairn, H., Mitchell, S., Robertson, L. D., Davidson, A., Ahmadian, N., et al. (2021). A comparison between support vector machine and water cloud model for estimating crop leaf area index. *Remote Sensing,**13*(7), 1–20. 10.3390/rs1307134836817948 10.3390/rs13071348

[CR41] Im, J., Park, S., Rhee, J., Baik, J., & Choi, M. (2016). Downscaling of AMSR-E soil moisture with MODIS products using machine learning approaches. *Environmental Earth Sciences,**75*(15), 1120. 10.1007/s12665-016-5917-6 10.1007/s12665-016-5917-6

[CR42] Itzerott, S., Hohmann, Christian Stender, V., Maass, H., Borg, E., Renke, F., Jahncke, D., et al. (2018). TERENO (Northeast), Climate stations of the GFZ German Research Centre for Geoscienes (GFZ). V. 2.0. GFZ Data Services. 10.5880/TERENO.GFZ.CL.2018.ALL

[CR43] Jackson, T. J., Cosh, M. H., Bindlish, R., Starks, P. J., Bosch, D. D., Seyfried, M., et al. (2010). Validation of advanced microwave scanning radiometer soil moisture products. *IEEE Transactions on Geoscience and Remote Sensing,**48*(12), 4256–4272. 10.1109/TGRS.2010.2051035 10.1109/TGRS.2010.2051035

[CR44] Jiménez-Muñoz, J. C., Sobrino, J. A., Plaza, A., Guanter, L., Moreno, J., & Martínez, P. (2009). Comparison between fractional vegetation cover retrievals from vegetation indices and spectral mixture analysis: Case study of PROBA/CHRIS data over an agricultural area. *Sensors,**9*(2), 768–793. 10.3390/s90200768 22399938 10.3390/s90200768 PMC3280830

[CR45] Ke, Y., Im, J., Park, S., & Gong, H. (2016). Downscaling of MODIS One kilometer evapotranspiration using Landsat-8 data and machine learning approaches. *Remote Sensing,**8*(3), 1–26. 10.3390/rs8030215 10.3390/rs8030215

[CR46] Kim, Y., & Van Zyl, J. (2004). Vegetation effects on soil moisture estimation. *International Geoscience and Remote Sensing Symposium (IGARSS),**2*, 800–802. 10.1109/IGARSS.2004.1368525 10.1109/IGARSS.2004.1368525

[CR47] Kim, Y., & Van Zyl, J. J. (2009). A time-series approach to estimate soil moisture using polarimetric radar data. *IEEE Transactions on Geoscience and Remote Sensing,**47*(8), 2519–2527. 10.1109/TGRS.2009.2014944 10.1109/TGRS.2009.2014944

[CR48] Kim, Y., Jackson, T., Bindlish, R., Lee, H., & Hong, S. (2012). Radar vegetation index for estimating the vegetation water content of rice and soybean. *IEEE Geoscience and Remote Sensing Letters,**9*(4), 564–568. 10.1109/LGRS.2011.2174772 10.1109/LGRS.2011.2174772

[CR49] Li, M., Sun, H., & Zhao, R. (2023). A review of root zone soil moisture estimation methods based on remote sensing. *Remote Sensing,**15*(22), 5361. 10.3390/RS15225361 10.3390/RS15225361

[CR50] Liaw, A., & Wiener, M. (2002). Classification and regression by random forest. *R News,**2*(3), 18–22.

[CR51] Liu, Y., Jing, W., Wang, Q., & Xia, X. (2020). Generating high-resolution daily soil moisture by using spatial downscaling techniques: A comparison of six machine learning algorithms. *Advances in Water Resources,**141*, 103601. 10.1016/j.advwatres.2020.103601 10.1016/j.advwatres.2020.103601

[CR52] Lv, A., Zhang, Z., & Zhu, H. (2021). A neural-network based spatial resolution downscaling method for soil moisture: Case study of Qinghai province. *Remote Sensing,**13*(8), 1–22. 10.3390/rs1308158336817948 10.3390/rs13081583

[CR53] Madelon, R., Rodríguez-Fernández, N. J., Bazzi, H., Baghdadi, N., Albergel, C., Dorigo, W., & Zribi, M. (2023). Soil moisture estimates at 1 km resolution making a synergistic use of Sentinel data. *Hydrology and Earth System Sciences,**27*(6), 1221–1242. 10.5194/hess-27-1221-2023 10.5194/hess-27-1221-2023

[CR54] Magdić, I., Safner, T., Rubinić, V., Rutić, F., Husnjak, S., & Filipović, V. (2022). Effect of slope position on soil properties and soil moisture regime of Stagnosol in the vineyard. *Journal of Hydrology and Hydromechanics,**70*(1), 62–73. 10.2478/johh-2021-0037 10.2478/johh-2021-0037

[CR55] Maggioni, V., Reichle, R. H., & Anagnostou, E. N. (2013). The efficiency of assimilating satellite soil moisture retrievals in a land data assimilation system using different rainfall error models. *Journal of Hydrometeorology,**14*(1), 368–374. 10.1175/JHM-D-12-0105.1 10.1175/JHM-D-12-0105.1

[CR56] Manfreda, S., Brocca, L., Moramarco, T., Melone, F., & Sheffield, J. (2014). A physically based approach for the estimation of root-zone soil moisture from surface measurements. *Hydrology and Earth System Sciences,**18*(3), 1199–1212. 10.5194/hess-18-1199-2014 10.5194/hess-18-1199-2014

[CR57] Martínez-Fernández, J., González-Zamora, A., & Almendra-Martín, L. (2021). Soil moisture memory and soil properties: An analysis with the stored precipitation fraction. *Journal of Hydrology,**593*, 125622. 10.1016/j.jhydrol.2020.125622 10.1016/j.jhydrol.2020.125622

[CR58] Merlin, O., Chehbouni, A., Walker, J. P., Panciera, R., & Kerr, Y. H. (2008). A simple method to disaggregate passive microwave-based soil moisture. *IEEE Transactions on Geoscience and Remote Sensing,**46*(3), 786–796. 10.1109/TGRS.2007.914807 10.1109/TGRS.2007.914807

[CR59] Merlin, O., Escorihuela, M. J., Mayoral, M. A., Hagolle, O., Al Bitar, A., & Kerr, Y. (2013). Self-calibrated evaporation-based disaggregation of SMOS soil moisture: An evaluation study at 3 km and 100 m resolution in Catalunya, Spain. *Remote Sensing of Environment,**130*, 25–38. 10.1016/J.RSE.2012.11.008 10.1016/J.RSE.2012.11.008

[CR60] Merzlyak, M. N., Gitelson, A. A., Chivkunova, O. B., & Rakitin, V. Y. (1999). Non-destructive optical detection of pigment changes during leaf senescence and fruit ripening. *Physiologia Plantarum,**106*(1), 135–141. 10.1034/J.1399-3054.1999.106119.X 10.1034/J.1399-3054.1999.106119.X

[CR61] Montzka, C., Rötzer, K., Bogena, H. R., Sanchez, N., & Vereecken, H. (2018). A new soil moisture downscaling approach for SMAP, SMOS, and ASCAT by predicting sub-grid variability. *Remote Sensing*, *10*(3). 10.3390/rs10030427

[CR62] Mullissa, A., Vollrath, A., Odongo-Braun, C., Slagter, B., Balling, J., Gou, Y., et al. (2021). Sentinel-1 SAR backscatter analysis ready data preparation in Google Earth Engine. *Remote Sensing,**13*(10), 5–11. 10.3390/rs13101954 10.3390/rs13101954

[CR63] Ojha, N., Merlin, O., Molero, B., Suere, C., Olivera-Guerra, L., Hssaine, B. A., et al. (2019). Stepwise disaggregation of SMAP soil moisture at 100 m resolution using Landsat-7/8 data and a varying intermediate resolution. *Remote Sensing,**11*(16), 1–23. 10.3390/rs11161863 10.3390/rs11161863

[CR64] Pablos, M., González-Zamora, Á., Sánchez, N., & Martínez-Fernández, J. (2018). Assessment of root zone soil moisture estimations from SMAP, SMOS and MODIS observations. *Remote Sensing,**10*(7), 981. 10.3390/rs10070981 10.3390/rs10070981

[CR65] Paulik, C., Dorigo, W., Wagner, W., & Kidd, R. (2014). Validation of the ASCAT soil water index using in situ data from the International Soil Moisture Network. *International Journal of Applied Earth Observation and Geoinformation,**30*(1), 1–8. 10.1016/j.jag.2014.01.007 10.1016/j.jag.2014.01.007

[CR66] Pawar, J., & Khanna, R. (2018). More crop per drop: Ways to increase water use efficiency for crop production: A review.* International Journal of Chemical Studies*, *6*(3), 3573–3578.

[CR67] Peng, J., Loew, A., Merlin, O., & Verhoest, N. E. C. (2017). A review of spatial downscaling of satellite remotely sensed soil moisture. *Reviews of Geophysics,**55*(2), 341–366. 10.1002/2016RG000543 10.1002/2016RG000543

[CR68] Piedallu, C., Gégout, J. C., Perez, V., & Lebourgeois, F. (2013). Soil water balance performs better than climatic water variables in tree species distribution modelling. *Global Ecology and Biogeography,**22*(4), 470–482. 10.1111/geb.12012 10.1111/geb.12012

[CR69] Piles, M., Camps, A., Vall-llossera, M., Sánchez, N., Martínez-Fernández, J., Monerris, A., et al. (2010). Soil moisture downscaling activities at the REMEDHUS Cal/Val site and its application to SMOS. In *2010 11th Specialist Meeting on Microwave Radiometry and Remote Sensing of the Environment* (pp. 17–21). 10.1109/MICRORAD.2010.5559599

[CR70] Prajapati, R., Chakraborty, D., & Kumar, V. (2018). Advances in soil moisture retrieval from near-surface measurements using satellite remote sensing. *The International Archives of the Photogrammetry, Remote Sensing and Spatial Information Sciences*, *XLII–5*(5), 861–869. 10.5194/ISPRS-ARCHIVES-XLII-5-861-2018

[CR71] Qu, Y., Zhu, Z., Montzka, C., Chai, L., Liu, S., Ge, Y., et al. (2021). Inter-comparison of several soil moisture downscaling methods over the Qinghai-Tibet Plateau, China. *Journal of Hydrology,**592*(October 2020), 125616. 10.1016/j.jhydrol.2020.125616 10.1016/j.jhydrol.2020.125616

[CR72] Raduła, M. W., Szymura, T. H., & Szymura, M. (2018). Topographic wetness index explains soil moisture better than bioindication with Ellenberg’s indicator values. *Ecological Indicators,**85*(October 2017), 172–179. 10.1016/j.ecolind.2017.10.011 10.1016/j.ecolind.2017.10.011

[CR73] Rasheed, M. W., Tang, J., Sarwar, A., Shah, S., Saddique, N., Khan, M. U., et al. (2022). Soil moisture measuring techniques and factors affecting the moisture dynamics: A comprehensive review. *Sustainability (Switzerland)*, *14*(18). 10.3390/su141811538

[CR74] Reichle, R. H., Liu, Q., Koster, R. D., Crow, W. T., De Lannoy, G. J. M., Kimball, J. S., et al. (2019). Version 4 of the SMAP level-4 soil moisture algorithm and data product. *Journal of Advances in Modeling Earth Systems,**11*(10), 3106–3130. 10.1029/2019MS001729 10.1029/2019MS001729

[CR75] Reuß, F., Navacchi, C., Greimeister-Pfeil, I., Vreugdenhil, M., Schaumberger, A., Klingler, A., et al. (2024). Evaluation of limiting factors for SAR backscatter based cut detection of alpine grasslands. *Science of Remote Sensing,**9*(October 2023), 100117. 10.1016/j.srs.2024.100117 10.1016/j.srs.2024.100117

[CR76] Tang, K., Zhu, H., & Ni, P. (2021). Spatial downscaling of land surface temperature over heterogeneous regions using random forest regression considering spatial features. *Remote Sensing,**13*(18), 3645. 10.3390/rs13183645 10.3390/rs13183645

[CR77] Taylor, K. E. (2001). Summarizing multiple aspects of model performance in a single diagram. *Journal of Geophysical Research, 106*, 7183-7192.

[CR78] Trudel, M., Charbonneau, F., & Leconte, R. (2012). Using RADARSAT-2 polarimetric and ENVISAT-ASAR dual-polarization data for estimating soil moisture over agricultural fields. *Canadian Journal of Remote Sensing,**38*(4), 514–527. 10.5589/M12-043 10.5589/M12-043

[CR79] Ustin, S. L., & Middleton, E. M. (2021). Current and near-term advances in Earth observation for ecological applications. *Ecological Processes,**10*(1), 1–57. 10.1186/s13717-020-00255-4 33425642 10.1186/s13717-020-00255-4 PMC7779249

[CR80] Wagner, W. (1998). *A method for estimating soil moisture from ERS scatterometer and soil data*. Vienna University of Technology. Retrieved from http://www.ncbi.nlm.nih.gov/pubmed/16366269

[CR81] Wagner, W., Lemoine, G., & Rott, H. (1999). A method for estimating soil moisture from ERS scatterometer and soil data. *Remote Sensing of Environment,**70*(2), 191–207. 10.1016/S0034-4257(99)00036-X 10.1016/S0034-4257(99)00036-X

[CR82] Wakigari, S. A., & Leconte, R. (2022). Enhancing spatial resolution of SMAP soil moisture products through spatial downscaling over a large watershed: A case study for the Susquehanna River Basin in the Northeastern United States. *Remote Sensing,**14*(3), 1–35. 10.3390/rs14030776 10.3390/rs14030776

[CR83] Wang, T., Franz, T. E., You, J., Shulski, M. D., & Ray, C. (2017). Evaluating controls of soil properties and climatic conditions on the use of an exponential filter for converting near surface to root zone soil moisture contents. *Journal of Hydrology,**548*, 683–696. 10.1016/j.jhydrol.2017.03.055 10.1016/j.jhydrol.2017.03.055

[CR84] Wang, Q., Tang, Y., Ge, Y., Xie, H., Tong, X., & Atkinson, P. M. (2023). A comprehensive review of spatial-temporal-spectral information reconstruction techniques. *Science of Remote Sensing,**8*, 100102. 10.1016/J.SRS.2023.100102 10.1016/J.SRS.2023.100102

[CR85] Wu, Z., Feng, H., He, H., Zhou, J., & Zhang, Y. (2021). Evaluation of soil moisture climatology and anomaly components derived from ERA5-Land and GLDAS-2.1 in China. *Water Resources Management,**35*(2), 629–643. 10.1007/s11269-020-02743-w 10.1007/s11269-020-02743-w

[CR86] Xing, M., Chen, L., Wang, J., Shang, J., & Huang, X. (2022). Soil moisture retrieval using SAR backscattering ratio method during the crop growing season. *Remote Sensing,**14*(13), 3210. 10.3390/rs14133210 10.3390/rs14133210

[CR87] Yang, Y., Bao, Z., Wu, H., Wang, G., Liu, C., Wang, J., & Zhang, J. (2022). An exponential filter model-based root-zone soil moisture estimation methodology from multiple datasets. *Remote Sensing,**14*(8), 1–22. 10.3390/rs14081785 10.3390/rs14081785

[CR88] Yu, X., Zhang, S., Li, J., Lu, L., Liu, Z., Li, M., et al. (2019). A multi-timescale EnOI-like high-efficiency approximate filter for coupled model data assimilation. *Journal of Advances in Modeling Earth Systems,**11*(1), 45–63. 10.1029/2018MS001504 10.1029/2018MS001504

[CR89] Zacharias, S., Bogena, H., Samaniego, L., Mauder, M., Fuß, R., Pütz, T., et al. (2011). A network of terrestrial environmental observatories in Germany. *Vadose Zone Journal,**10*(3), 955–973. 10.2136/VZJ2010.0139 10.2136/VZJ2010.0139

[CR90] Zanaga, D., Van De Kerchove, R., De Keersmaecker, W., Souverijns, N., Brockmann, C., Quast, R., et al. (2021). Land cover (Global - 10m–2021) - ESA WorldCover - Datasets. https://worldcover2021.esa.int/

[CR91] Zawadzki, J., & Kędzior, M. (2016). Soil moisture variability over Odra watershed: Comparison between SMOS and GLDAS data. *International Journal of Applied Earth Observation and Geoinformation,**45*, 110–124. 10.1016/J.JAG.2015.03.005 10.1016/J.JAG.2015.03.005

[CR92] Zhao, W., Li, A., Jin, H., Zhang, Z., Bian, J., & Yin, G. (2017). Performance evaluation of the triangle-based empirical soil moisture relationship models based on Landsat-5 TM data and in situ measurements. *IEEE Transactions on Geoscience and Remote Sensing,**55*(5), 2632–2645. 10.1109/TGRS.2017.2649522 10.1109/TGRS.2017.2649522

[CR93] Zheng, Y., Coxon, G., Woods, R., Power, D., & Rosolem, R. (2022). Evaluation of reanalysis soil moisture products using cosmic ray neutron sensor observations across the globe, (October), 1–27. 10.5194/egusphere-egu22-3206

[CR94] Zhu, S., Wang, X., Jiao, D., Zhang, Y., & Liu, J. (2023). Spatial downscaling of GPM satellite precipitation data using extreme random trees. *Atmosphere,**14*(10), 1489. 10.3390/atmos14101489 10.3390/atmos14101489

