# Supplementary material for: Estimation of 100 m root zone soil moisture by downscaling 1 km soil water index with machine learning and multiple geodata
Source: Environ Monit Assess. 2024 Aug 19;196(9):823. doi: 10.1007/s10661-024-12969-5 (PMC11333521; doi:10.1007/s10661-024-12969-5)
Supplement: Supplementary file 1 — Supplementary Material 1 [file 10661_2024_12969_MOESM1_ESM.docx]

**Supplementary Material**

**Table S1.** RF1, RF2 and RF3 predicted SWI_T-100m_ based on Sentinel-1 and Sentinel-2 dates

| **RF1** | **RF2** | **RF3** |
| --- | --- | --- |
| 03/01/2018 | 07/01/2018 | 05/01/2018 |
| 09/01/2018 | 01/02/2018 | 02/02/2018 |
| 15/01/2018 | 06/02/2018 | 04/02/2018 |
| 21/01/2018 | 16/02/2018 | 16/02/2018 |
| 27/01/2018 | 03/03/2018 | 04/03/2018 |
| 02/02/2018 | 02/04/2018 | 03/04/2018 |
| 08/02/2018 | 17/04/2018 | 17/04/2018 |
| 14/02/2018 | 27/04/2018 | 27/04/2018 |
| 20/02/2018 | 07/05/2018 | 05/05/2018 |
| 26/02/2018 | 17/05/2018 | 17/05/2018 |
| 04/03/2018 | 22/05/2018 | 21/05/2018 |
| 10/03/2018 | 06/06/2018 | 04/06/2018 |
| 16/03/2018 | 26/06/2018 | 26/06/2018 |
| 28/03/2018 | 01/07/2018 | 02/07/2018 |
| 03/04/2018 | 06/07/2018 | 04/07/2018 |
| 09/04/2018 | 16/07/2018 | 16/07/2018 |
| 15/04/2018 | 21/07/2018 | 20/07/2018 |
| 21/04/2018 | 26/07/2018 | 26/07/2018 |
| 27/04/2018 | 31/07/2018 | 01/08/2018 |
| 03/05/2018 | 04/09/2018 | 02/09/2018 |
| 09/05/2018 | 09/09/2018 | 08/09/2018 |
| 15/05/2018 | 14/09/2018 | 14/09/2018 |
| 21/05/2018 | 19/09/2018 | 18/09/2018 |
| 27/05/2018 | 24/09/2018 | 24/09/2018 |
| 02/06/2018 | 29/09/2018 | 30/09/2018 |
| 08/06/2018 | 14/10/2018 | 14/10/2018 |
| 14/06/2018 | 19/10/2018 | 18/10/2018 |
| 20/06/2018 | 24/10/2018 | 24/10/2018 |
| 26/06/2018 | 03/11/2018 | 01/11/2018 |
| 02/07/2018 | 28/11/2018 | 29/11/2018 |
| 08/07/2018 |  |  |
| 14/07/2018 |  |  |
| 20/07/2018 |  |  |
| 26/07/2018 |  |  |
| 01/08/2018 |  |  |
| 07/08/2018 |  |  |
| 13/08/2018 |  |  |
| 19/08/2018 |  |  |
| 25/08/2018 |  |  |
| 31/08/2018 |  |  |
| 06/09/2018 |  |  |
| 12/09/2018 |  |  |
| 18/09/2018 |  |  |
| 24/09/2018 |  |  |
| 30/09/2018 |  |  |
| 06/10/2018 |  |  |
| 12/10/2018 |  |  |
| 18/10/2018 |  |  |
| 24/10/2018 |  |  |
| 30/10/2018 |  |  |
| 05/11/2018 |  |  |
| 11/11/2018 |  |  |
| 17/11/2018 |  |  |
| 23/11/2018 |  |  |
| 29/11/2018 |  |  |
| 05/12/2018 |  |  |
| 11/12/2018 |  |  |
| 17/12/2018 |  |  |
| 23/12/2018 |  |  |
| 29/12/2018 |  |  |
| 05/01/2018 |  |  |
| 11/01/2018 |  |  |
| 17/01/2018 |  |  |
| 23/01/2018 |  |  |
| 29/01/2018 |  |  |
| 04/02/2018 |  |  |
| 10/02/2018 |  |  |
| 16/02/2018 |  |  |
| 22/02/2018 |  |  |
| 28/02/2018 |  |  |
| 06/03/2018 |  |  |
| 12/03/2018 |  |  |
| 18/03/2018 |  |  |
| 24/03/2018 |  |  |
| 30/03/2018 |  |  |
| 05/04/2018 |  |  |
| 11/04/2018 |  |  |
| 17/04/2018 |  |  |
| 23/04/2018 |  |  |
| 29/04/2018 |  |  |
| 05/05/2018 |  |  |
| 11/05/2018 |  |  |
| 17/05/2018 |  |  |
| 23/05/2018 |  |  |
| 29/05/2018 |  |  |
| 04/06/2018 |  |  |
| 10/06/2018 |  |  |
| 16/06/2018 |  |  |
| 22/06/2018 |  |  |
| 28/06/2018 |  |  |
| 04/07/2018 |  |  |
| 10/07/2018 |  |  |
| 16/07/2018 |  |  |
| 22/07/2018 |  |  |
| 28/07/2018 |  |  |
| 03/08/2018 |  |  |
| 09/08/2018 |  |  |
| 15/08/2018 |  |  |
| 21/08/2018 |  |  |
| 27/08/2018 |  |  |
| 02/09/2018 |  |  |
| 08/09/2018 |  |  |
| 14/09/2018 |  |  |
| 20/09/2018 |  |  |
| 26/09/2018 |  |  |
| 02/10/2018 |  |  |
| 08/10/2018 |  |  |
| 14/10/2018 |  |  |
| 20/10/2018 |  |  |
| 26/10/2018 |  |  |
| 01/11/2018 |  |  |
| 07/11/2018 |  |  |
| 13/11/2018 |  |  |
| 19/11/2018 |  |  |
| 25/11/2018 |  |  |
| 01/12/2018 |  |  |
| 07/12/2018 |  |  |
| 13/12/2018 |  |  |
| 19/12/2018 |  |  |
| 25/12/2018 |  |  |
| 31/12/2018 |  |  |


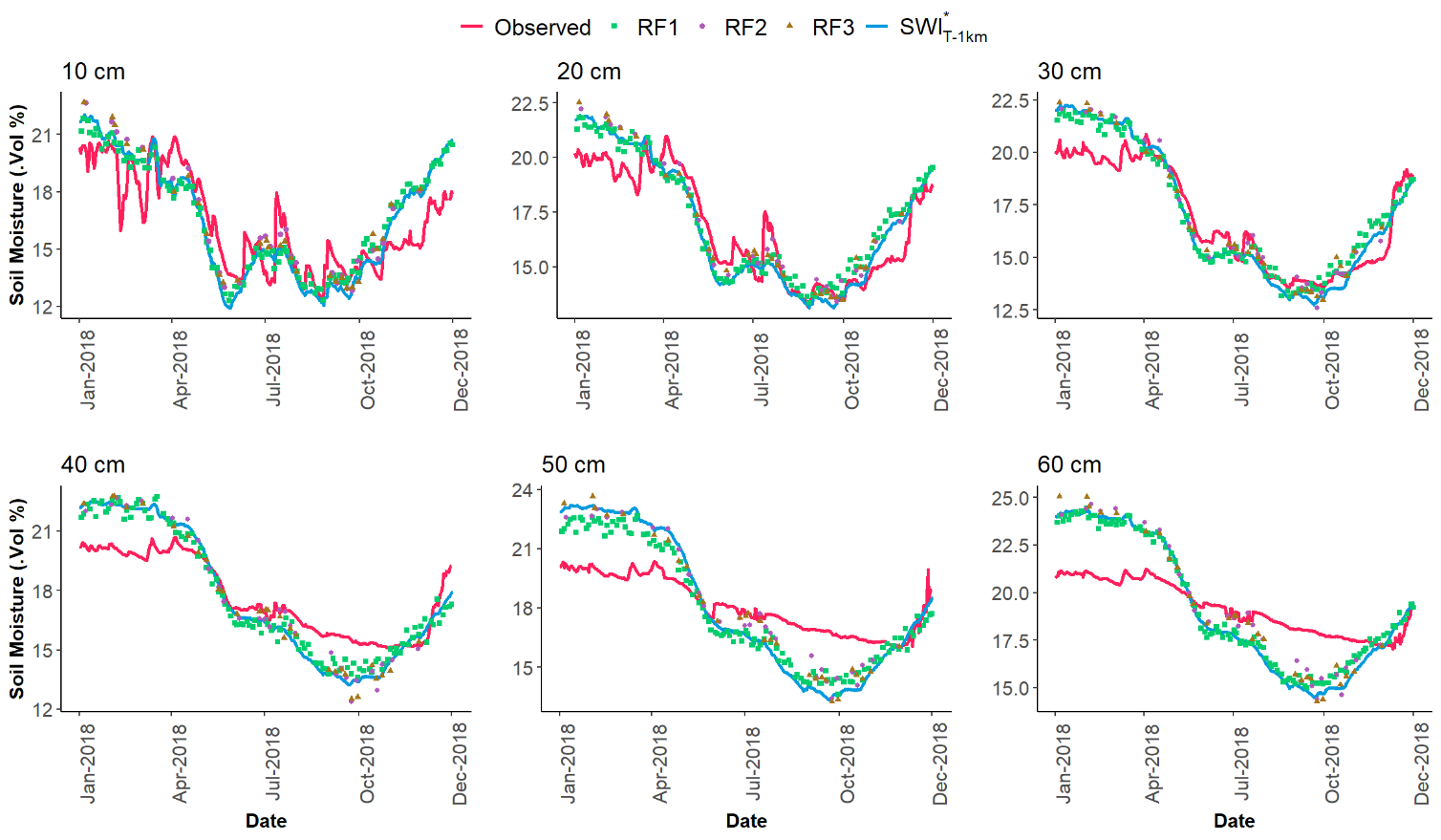


**Figure S1**: Average comparison between in-situ RZSM and SWI^*^_T_ (1 km and 100 m) with selected T_opt_. RZSM: root zone soil moisture; SWI^*^_T-1km:_ converted 1 km SWI dataset; SWI^*^_T-100m_: converted downscaled 100 m SWI; T_opt_: optimized time length

.
